# Prioritization of Early-Stage Research and Development of a Hydrogel-Encapsulated Anaerobic Technology for Distributed Treatment of High Strength Organic Wastewater

**DOI:** 10.1021/acs.est.4c05389

**Published:** 2024-10-26

**Authors:** Xinyi Zhang, William A. Arnold, Natasha Wright, Paige J. Novak, Jeremy S. Guest

**Affiliations:** † Department of Civil and Environmental Engineering, University of Illinois Urbana–Champaign, 3221 Newmark Civil Engineering Laboratory, 205 N. Mathews Avenue, Urbana, Illinois 61801, United States; ‡ Department of Civil, Environmental, and Geo- Engineering, University of Minnesota, 500 Pillsbury Drive S.E., Minneapolis, Minnesota 55455, United States; § Department of Mechanical Engineering, University of Minnesota, 111 Church Street SE, Minneapolis, Minnesota 55455, United States; ∥ Institute for Sustainability, Energy, and Environment, University of Illinois Urbana–Champaign, 1101 W. Peabody Drive, Urbana, Illinois 61801, United States; ⊥ DOE Center for Advanced Bioenergy and Bioproducts Innovation, University of Illinois Urbana–Champaign, 1206 W. Gregory Drive, Urbana, Illinois 61801, United States

**Keywords:** hydrogel encapsulation, biomass immobilization, anaerobic treatment, biogas recovery, greenhouse gas (GHG) emissions, quantitative sustainable design

## Abstract

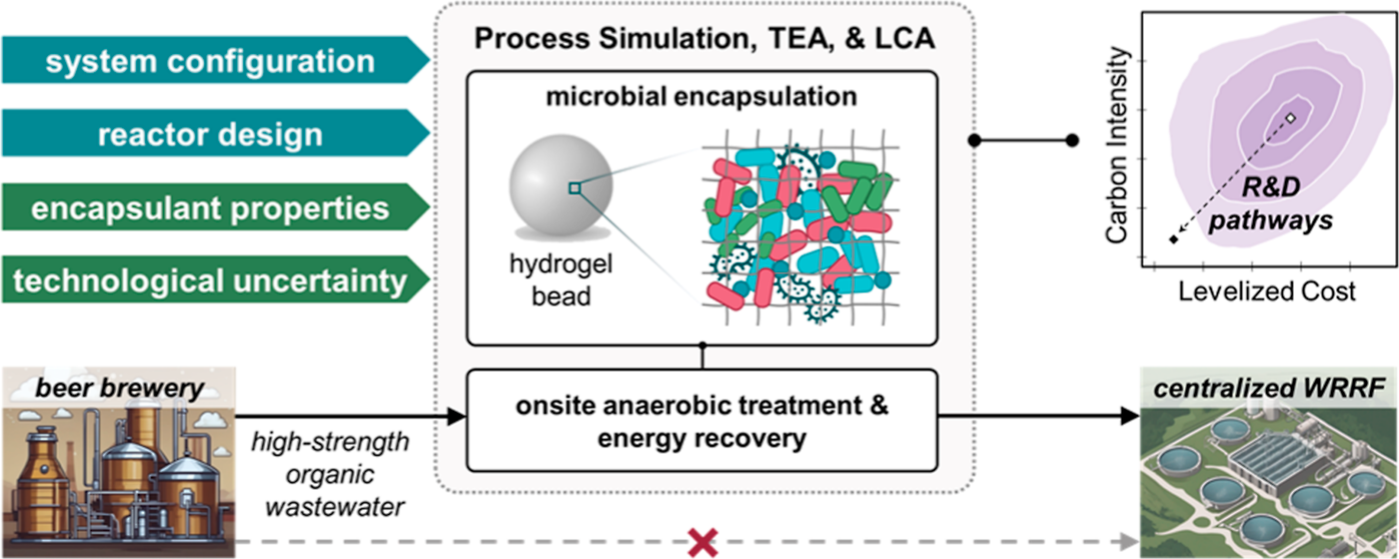

This study aims to support the prioritization of research and development (R&D) pathways of an anaerobic technology leveraging hydrogel-encapsulated biomass to treat high-strength organic industrial wastewaters, enabling decentralized energy recovery and treatment to reduce organic loading on centralized treatment facilities. To characterize the sustainability implications of early-stage design decisions and to delineate R&D targets, an encapsulated anaerobic process model was developed and coupled with design algorithms for integrated process simulation, techno-economic analysis, and life cycle assessment under uncertainty. Across the design space, a single-stage configuration with passive biogas collection was found to have the greatest potential for financial viability and the lowest life cycle carbon emission. Through robust uncertainty and sensitivity analyses, we found technology performance was driven by a handful of design and technological factors despite uncertainty surrounding many others. Hydraulic retention time and encapsulant volume were identified as the most impactful design decisions for the levelized cost and carbon intensity of chemical oxygen demand (COD) removal. Encapsulant longevity, a technological parameter, was the dominant driver of system sustainability and thus a clear R&D priority. Ultimately, we found encapsulated anaerobic systems with optimized fluidized bed design have significant potential to provide affordable, carbon-negative, and distributed COD removal from high strength organic wastewaters if encapsulant longevity can be maintained at 5 years or above.

## Abstract 2

Targeted research and development on hydrogel-encapsulated microbial consortia can support decentralized bioenergy production, while reducing the burden on centralized water resource recovery facilities.

## Introduction

Biomass immobilization has been investigated for decades as a technique to enhance process performance in various fields of biotechnology.^1^ By fixing or stabilizing biomass onto or within a support material, this technique simplifies the separation of biomass from the reaction mixture, facilitating recovery and reuse of biomass and improving volumetric productivity.^2^ Among common immobilization methods, encapsulation or entrapment with hydrogel has attracted increasing research interests in its environmental applications: for example, the use of enzyme biocatalysts for emerging contaminant removal,^3,4^ pure or mixed culture for nutrient removal,^5−9^ microalgae for phosphorus recovery,^10^ and sludge for biohydrogen production^11−13^ or anaerobic treatment of organic waste streams.^14,15^ Hydrogel encapsulation or entrapment offer distinct advantages, such as concentrating specific biomass types,^16^ improving process resilience to environmental stresses like fluctuating pH or inhibitor accumulation,^16,17^ and allowing biomass preparation to be independent from the system start-up and long-term operation.^2^ As a result, biomass encapsulation technology holds unique potential for decentralized wastewater treatment and resource recovery in small- or medium-sized industrial settings, where fast start-up, reliable treatment of highly variable waste streams, and ease of operation are critical concerns.

One potential application of encapsulant technologies is the distributed treatment of high-strength aqueous waste organics, which represent both a challenge for centralized water resource recovery facilities (WRRFs)^18,19^ and an opportunity to support industrial decarbonization (a.k.a. defossilization).^20,21^ The food and beverage industry, in particular, is one of the five most carbon-intensive manufacturing subsectors in the United States.^22^ Food and beverage industry wastewaters frequently have concentrated organics (e.g., 1 to 10 g-COD·L^–1^)^23^ that are often treated aerobically in centralized WRRFs at an energy demand of 0.4–1.2 kW h·kg^–1^ COD removed.^24,25^ As an alternative, anaerobic biotechnology has the potential to convert the waste organics into bioenergy^26−28^ and/or high-value bioproducts (e.g., medium-chain fatty acids^29−31^). Due to the slower growth rate of anaerobic microorganisms, supporting technologies that decouple solids residence time from hydraulic retention time (HRT), including biomass immobilization, have been identified as key research foci for cost-effective and energy-efficient applications at a small or medium scale.^32^ Despite the extensive research on anaerobic treatment of industrial waste streams and the accumulating knowledge of encapsulation chemistry, the implications of microbial encapsulation on anaerobic process kinetics, design, and operational requirements, and ultimately the life cycle cost and environmental impacts of such treatment systems are still highly uncertain.

To guide the research and development (R&D) of encapsulated anaerobic technologies, models are needed to simulate the effects of wastewater composition, encapsulation matrix properties, and reactor design and operation on treatment performance as well as the input and output flows of the system throughout its life cycle. Work has been done to understand certain aspects of treatment performance in response to design and operating conditions of encapsulated biological systems. For example, Zhu et al. parametrized the effects of changing HRT, bead size, and feed substrate concentration on the hydrogen production rate of alginate-encapsulated biomass based on a classic diffusion-reaction model.^33^ Wang et al. modified a 1-D biofilm model to describe encapsulated growth of ammonia oxidizing bacteria and enabled optimizations of critical design decisions of the encapsulation matrix for nitrogen removal.^34^ Although it has been recognized that the use of encapsulant materials—especially petroleum-based hydrogels (e.g., poly(vinyl alcohol),^35^ waterborne polyurethane,^6,36^ and polyethylene glycol (PEG)^5^)—can affect system sustainability in complicated ways,^8,37^ there is still a lack of understanding of how individual design decisions and technology performance parameters are likely to impact the net cost and life cycle environmental impacts. Thus far, quantitative discussions to guide the R&D of encapsulated biological treatment systems have generally focused on improving treatment efficacy. To better inform the early-stage R&D of encapsulation technology, specifically for its industrial application in distributed anaerobic treatment of high strength wastewater, it is imperative to computationally couple process modeling with rigorous economic and environmental impact analyses,^38^ so we can understand how individual decision variables (e.g., reactor design, single-stage vs two-stage configuration) and technological uncertainty (e.g., biomass encapsulation capacity and encapsulant durability) drive system-level financial viability and environmental sustainability.

The objective of this work was to characterize the potential financial and environmental implications of distributed anaerobic treatment of high-strength organic industrial wastewater using encapsulated biomass. By assessing a range of design decisions and technological assumptions, opportunities to improve cost and environmental outcomes were identified and prioritized for R&D investment. To achieve this outcome, we developed a computational model for process simulation and design of encapsulated anaerobic systems with a focus on hydrogen (H_2_) and methane (CH_4_) production. The financial viability and environmental sustainability of applying encapsulated systems for onsite treatment of brewery wastewater were evaluated through techno-economic analysis (TEA) and life cycle assessment (LCA) and benchmarked against conventional upflow anaerobic sludge blanket (UASB) systems. Uncertainty in design and performance were considered in a Monte Carlo simulation framework. The relative impacts of individual design decisions and technological assumptions on system sustainability were quantified through robust global sensitivity analyses. Finally, quantitative recommendations were provided for R&D prioritization of encapsulated anaerobic technologies.

## Methods

**Figure 1:**
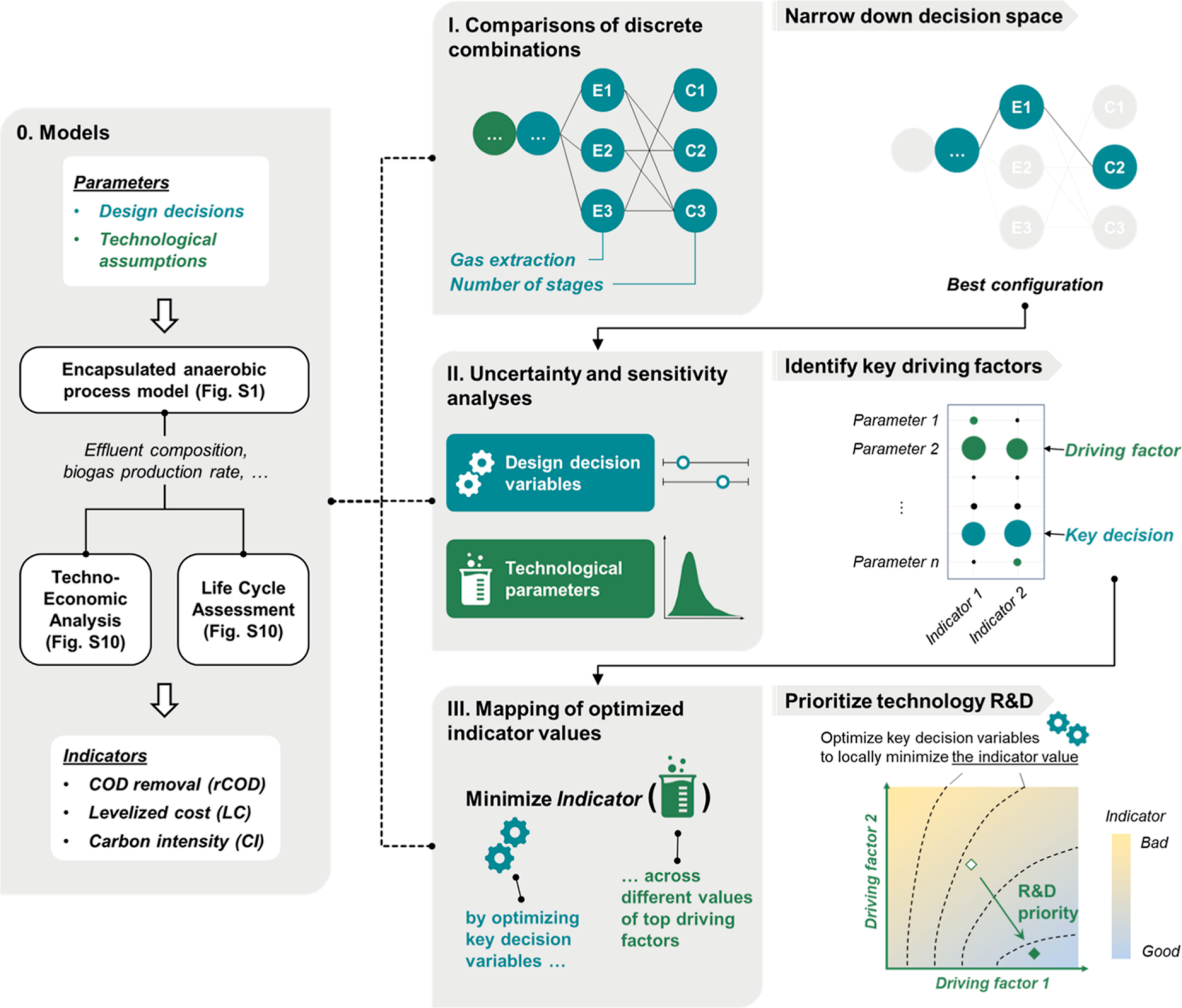
Illustration of the modeling and analysis framework used in this study. Solid arrows indicate the order in which the stages of the analysis were performed as well as the flows of information. Thick white arrows indicate model inputs and outputs. Dotted lines indicate the models are used consistently throughout the three stages of analyses.

We centered our analysis on an encapsulated anaerobic technology targeting onsite treatment of and energy recovery (via H_2_ and CH_4_) from a brewery’s wastewater prior to discharge to a centralized conventional treatment facility located in St. Paul, Minnesota. The brewery produces 50 m^3^·d^–1^ of high-strength wastewater with a total COD of 6760 mg·L^–1^ and a soluble COD of 5640 mg·L^–1^ on average.^39^ To evaluate treatment performance (i.e., COD removal) and system sustainability (i.e., economic and environmental indicators) across a broad design landscape, we employed the quantitative sustainable design (QSD) methodology integrating process simulation, system design, TEA, and LCA under uncertainty across three stages of analysis (Figure 1).^40^ The implementation of this approach was facilitated by the Python package QSDsan.^41^ All source code for modeling, simulation, and assessment of the system can be found in the open-access Python repository EXPOsan.^42^

### Process Model, System Design, TEA, and LCA

The system is mainly composed of either a single-stage or a two-stage encapsulated anaerobic reactor and optional auxiliary unit operations, such as degassing membrane contactor, iron sponge scrubber, and double-membrane biogas holder. A two-stage system consists of a fermenting first stage and a methanogenic second stage, which differ in the initial relative abundance of acidogens, acetogens, and methanogens within the encapsulation matrix besides pH (Section S4). The biogas from the anaerobic system is assumed to be reused for heating onsite at the brewery, taking advantage of its existing infrastructure (i.e., the natural gas boiler and heat exchangers) and offsetting natural gas purchases. For benchmarking purposes, UASB reactors were also modeled to represent the performance of state-of-the-art anaerobic technologies without encapsulation.

#### Process Model

A process model was developed and verified with batch experimental data to establish dynamic connections between system design and treatment performance by considering a series of physicochemical and biological processes in an encapsulated anaerobic environment (Section S1). Decision variables and technological assumptions were input into the process model for simulations of the mass and energy balances in the system. After converging to a steady state, the model was used to translate the mass flow data of the simulated system’s effluent and biogas streams into indicators of treatment performance, such as a COD removal percentage (rCOD, defined as the percent difference between the system effluent COD and influent brewery wastewater COD) and CH_4_ production rate. Identical assumptions about anaerobic biochemical processes were applied to the simulations of UASB systems, which mainly differ from encapsulated systems in reactor hydrodynamic and mass transport properties.

#### System Design

All reactor vessels were assumed to be cylindrical and constructed using concrete with rockwool for insulation and a thin carbon steel exterior facing. The UASB reactor also included stainless-steel three-phase separators. PEG was assumed to be the main encapsulant material.^43^ Hollow-fiber membrane contactors could be applied to remove dissolved CH_4_ from the effluent and/or to actively extract dissolved H_2_ from an externally recirculating sidestream of the first-stage reactor, depending on the system configuration.^44^ High density polyethylene pipes were used for liquid influent and effluent streams, whereas stainless-steel pipes were assumed for biogas streams. Equipment, such as water pumps, vacuum pumps, air compressors, heat exchangers, and control systems, were all included within the system boundary when applicable (Figure S10). The detailed design and costing algorithms of all unit operations and equipment can be found in Section S2 of Supporting Information.

#### TEA

Using the system boundary described in Figure S10, costs for the construction, operation, and maintenance (O&M) of unit operations were calculated using equations detailed in Section S2. The calculated costs and revenue were leveraged in a discounted cash flow analysis with QSDsan’s *TEA* class.^45^ To enable intersystem comparison, the levelized cost of COD removal (LC; defined as negative of annualized net present value divided by annual COD removal, in USD·tonne^–1^ COD removed) was calculated assuming a constant 5% discount rate and a 30 year project lifetime for all configurations. All monetary values were adjusted to 2021 US dollars.

#### LCA

Using the same system boundary described above, LCA was carried out to quantify the life cycle environmental impacts of the system following the general methodology outlined in ISO 14040/14044.^46,47^ For consistency with TEA, 1 tonne of COD removal was chosen as the functional unit of the analysis. While construction and O&M of all unit operations and equipment were included in the system boundary, project end-of-life was excluded due to lack of information about encapsulated systems. With the construction material, equipment, and O&M inputs and outputs (e.g., chemical use, electricity consumption, heat utility, bead replacement, and fugitive emissions) estimated through system simulations and the design algorithms, the corresponding life cycle inventory data and impact factors were gathered from the ecoinvent v3.8 database.^48^ Surrogate items or items in upstream production processes were used when a particular item was not available in the database (Table S2). The life cycle impact assessment was conducted using the tool for the reduction and assessment of chemical and other environmental impacts (TRACI v2.1).^49^ All nine impact categories evaluated by TRACI v2.1 were included in the simulations, with emphasis on the 100 year global warming potential to represent carbon intensity (CI) in subsequent analyses.

### Identifying Key Drivers for System Sustainability

#### Stage I. Discrete Decision Analysis

Given the early stage of encapsulated anaerobic system R&D, the most promising system configuration remains highly uncertain. To explore the broad landscape of possible designs for high strength wastewater distributed treatment and resource recovery, we evaluated 3552 distinct combinations of 11 design or operation decisions (Figure S11) and determined the LC and life cycle environmental impacts of COD removal. We considered four discrete decision variables: reactor type (fluidized bed or packed bed),^11^ number of stages (single-stage vs two-stage),^50,51^ H_2_ extraction from the first-stage reactor (passive collection, vacuum extraction from the reactor headspace, or sidestream membrane extraction), and whether to include a degassing membrane contactor for effluent methane management.^52^ We also varied seven continuous parameters of significance in early stage R&D.^11,53,54^ The parameter values were chosen to span common ranges observed in the cited studies or used in our experiments. A wider range was used if the parameter was deemed highly uncertain due to the limited information found in the literature. Two distinct external recirculation ratios (1 or 50) were considered for systems adopting sidestream membrane extraction of H_2_ and two distinct vacuum pressures (0.1, 0.4 bar) for vacuum extraction from headspace.^44^ For systems with encapsulated biomass, three discrete bead sizes (2, 5, and 10 mm)^12,16,55−57^ and three distinct bead lifetimes (1, 10, or 30 years)^7^ were considered in simulations. In addition, systems with fluidized beds were evaluated at three different bead volume fractions (0.10, 0.25, and 0.40). Reaction temperature (22 or 35 °C)^58^ and total HRT (1, 2, 4, or 12 days) were varied for all system configurations. Detailed simulation settings for all system configurations can be found in Section S4.

To quantify the relative impact of individual design decisions on system cost and CI, pairwise comparisons were conducted. For each decision variable, a baseline for comparison was first chosen (e.g., UASB as the baseline reactor type), and other values were considered alternatives (e.g., packed bed and fluidized bed for reactor type). All of the evaluated samples were organized into baseline-alternative pairs, each of which differs by only one common decision variable. A decision variable’s relative impact on a sustainability indicator (Δ*Y*, unitless) was calculated as the difference in the indicator values (i.e., *Y* being LC or CI) between a baseline–alternative pair normalized by the entire range of the indicator observed across 3552 distinct combinations (eq 1).
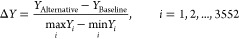
1

#### Stage II. Uncertainty and Sensitivity Analyses

The discrete decision analysis above can help identify the best performing designs and exclude unimpactful variables from subsequent analyses. While the previous analysis covered a broad design landscape, a more sophisticated variation of important continuous decision variables and technological uncertainties need to be incorporated in a rigorous uncertainty and sensitivity analysis to better inform decision making in the R&D of the encapsulated anaerobic biological technology. Therefore, we identified 18 independent parameters with uncertainty, including select decision variables that were found to be key drivers of system sustainability (e.g., HRT) in stage I analysis, 6 ADM1 kinetic parameters with a significant impact on COD removal,^59^ and a series of configuration-specific parameters characterizing the technological uncertainty (e.g., maximum encapsulation density, bead lifetime). The uncertainty of each parameter was characterized by a probability distribution derived from literature data or expert judgment (Table S5). All decision variables have a uniform distribution, representing full control within a feasible or desirable range from a technology developer’s perspective.

We performed a Monte Carlo simulation with Latin Hypercube Sampling^60^ (*N* = 1000) for each reactor type to propagate the uncertainty or variability of the 18 parameters. An identical set of samples was used across the three reactor types to enable pairwise comparisons. To elucidate the relative importance of different variables to the sustainability of encapsulated systems, we conducted Monte Carlo filtering^61^ for 5 indicators (i.e., rCOD, and LCs and CIs for COD removal with and without effluent degassing) using simulation data from the uncertainty analysis. Samples were divided into two groups, the top 25% (“desirable”) and the bottom 75% (“undesirable”)—based on the indicator value. For example, samples with rCOD higher than the 75th percentile were categorized into the “desirable” group, whereas for LC, samples lower than the 25th percentile were considered desirable. Two-sample Kolmogorov–Smirnov (KS) tests were used to characterize the difference in parameter distributions between the two groups and indicate whether a parameter, among others, plays a statistically significant role in yielding a desirable performance for an encapsulated system. The value of the KS test statistics *D* represents the “distance” between the parameter distributions of two sample groups. A larger *D* value suggests that the parameter plays a more important role (relative to other parameters) in yielding desirable outcomes. The *p*-value indicates the statistical (in-) significance of *D* > 0. To identify impactful factors for reactor choice, another KS test was performed between two groups of samples: when fluidized bed outperforms packed bed systems vs the opposite.

#### Stage III. Mapping the Critical Pathways for Technology R&D

We leveraged the uncertainty and sensitivity analyses above to identify key drivers for the economic and environmental sustainability of an encapsulated system. These driving factors are either decision variables (e.g., HRT), which can be readily optimized upon system design, or technological uncertainty (e.g., bead lifetime), which relies on technological advancement to attain desirable values. To characterize the sustainability frontier and to quantitatively delineate targets for technology R&D, the encapsulated systems were simulated across the two-dimensional space of pairs of key uncertain parameters identified above with other parameters fixed at their baseline values. For each uncertain parameter, grid samples were drawn from its defined range of uncertainty (i.e., 180 samples were evaluated for each pair of key uncertain parameters). For each sample, a bounded global optimization was performed to find the best values for decision variables (DVs) with a single objective to minimize the CI of COD removal (eq 2), given that a strong correlation between LC and CI had been observed for these encapsulated systems in the previous uncertainty and sensitivity analyses (Tables S6 and S7).
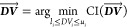
2

## Results and Discussion

### Stage I. Relative Impacts of Individual Design Decisions

Simulated performance and system sustainability varied widely across the 3552 distinct combinations of decision variables and technological assumptions. Simulated steady-state rCOD varied from 14.5% to 93.8% across designs, with the 5th and 95th percentiles being 35.7% and 85.7%, respectively. Projected LC and CI had right-tailed distributions spanning 32.4 to 81,363 USD·tonne^–1^ COD removed and −80.0 to 18,347 kg of CO_2_eq·tonne^–1^ COD removed, respectively (Figure S12). Only 108 out of 3552 designs were able to achieve negative CI for COD removal, whose LCs ranged from 32.4 to 848 USD·tonne^–1^ COD removed, lower than 65% of the evaluated designs. Therefore, specific combinations of design decisions have synergistic benefits for both financial viability and environmental sustainability.

**Figure 2:**
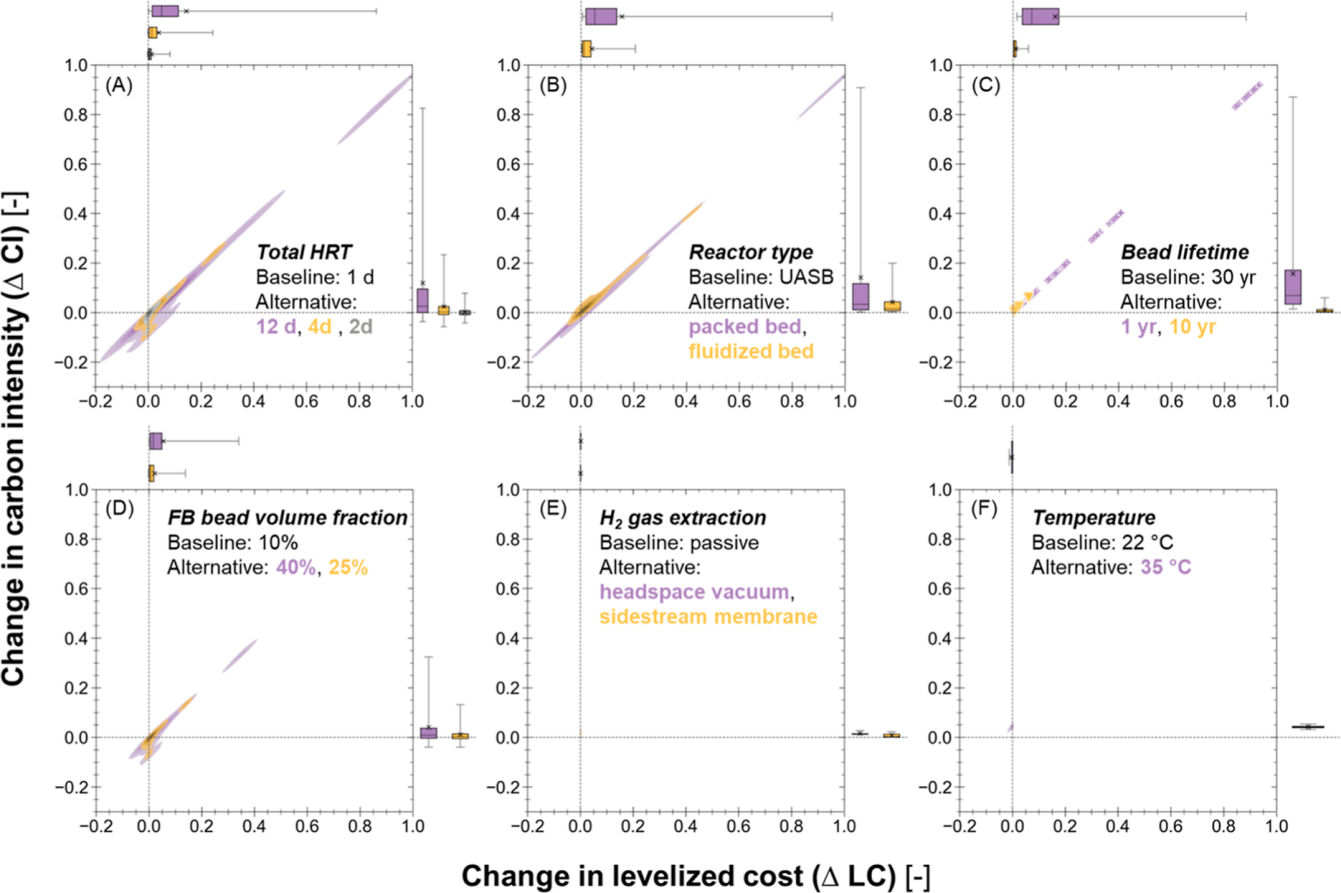
Kernel density (A,B,D,E,F) and scatter (C) plots of the relative impacts of individual design decisions on the LC and CI of COD removal. Definition of ΔLC and ΔCI follows eq 1. (C) Scatter plot rather than a kernel density plot is used to visualize the impacts of bead lifetime on LC and CI due to perfect linearity between the relative impacts on two metrics (bead lifetime directly impacted these two metrics via the exact same mechanism—bead replacement). Different alternative decisions are indicated by colors. Shades represent the estimated kernel density for a given alternative. Horizontal and vertical box-and-whisker plots illustrate the marginal distributions of ΔLC and ΔCI, respectively, sharing the *X*- and *Y*-axes with the main plots. In a box-and-whisker plot, the box extends from the 25th percentile to the 75th percentile of the data, with a line at the median and a marker (×) at the mean. The whiskers indicate the 5th and 95th percentiles of the data.

Among the 11 decision variables, total HRT and reactor type had the greatest relative impacts on both LC and CI (Figure 2). Under identical conditions within the evaluated ranges, UASB systems tended to have higher rCOD than encapsulated systems (by 2.9%–58.8% absolute difference to fluidized bed and −5.0% to 11.8% to packed bed) and was generally predicted to outperform them both economically and environmentally (Figure 2B). However, UASBs often require skilled labor for operation and their performance can be sensitive to changes in organic loading due to influent fluctuations:^62^ this has limited onsite deployment of UASBs at small- or medium-scale industries.^63,64^ As a result, although a UASB offers a useful technological comparison point, it may not be deployable or operable at the scale targeted by many encapsulation systems. The evaluated advantages of UASBs over encapsulated systems could be reduced or eliminated if costs of skilled labor or impacts associated with unstable treatment performance were parametrized in the models. However, such costs and impacts are highly dependent on the deployment context and are thus beyond the scope of our analysis.

Between encapsulated systems, packed bed systems tended to have higher predictions of rCOD but were usually subjected to higher LC and higher CI per tonne COD removed than a comparable fluidized bed system. Increasing total HRT from 1 to 4 days or above generally led to higher cost and impacts per tonne COD removed, with the increase in rCOD overshadowed by the quickly rising cost and impacts from the construction and O&M of a larger reactor (Figure 2A). Between 1 and 2 day HRTs, the implication was more nuanced. For example, increasing the HRT of a single-stage UASB system from 1 to 2 days reduced the CI per tonne of COD removed but raised the LC. For a single-stage fluidized bed system, however, a 2 day HRT could have both lower cost and lower impacts because further reducing HRT to 1 day was detrimental to COD removal performance.

For all encapsulated systems, the bead lifetime was a significant driver for LC and CI (Figure 2C). Shorter bead longevities resulted in higher bead replacement frequencies (e.g., 30 times throughout the 30 yr project lifetime with a 1 year longevity). The relative impacts of bead lifetime on LC and CI also scale with the amount of beads required. Therefore, the highest cost and impacts were observed with packed bed systems with long HRT and short bead lifetimes, and increasing the volume fraction of beads in a fluidized bed system tended to negatively affect its sustainability (Figure 2D).

Employing active H_2_ extraction or a mesophilic reactor temperature (35 °C), compared to passive collection or ambient temperature (22 °C), was found to have marginal impacts on LC but significantly increase CI (Figure 2E,F). This is because the improvements in rCOD (an absolute difference of −0.11% to 0.38% for active H_2_ extraction; 0.42%–29% for mesophilic temperatures) did not outweigh the additional cost and environmental impacts incurred from the installation and operation of vacuum pumps or membrane contactors and heat exchangers. Similar findings were reported in a previous study of an anaerobic system with immobilized biomass, where no significant difference in rCOD was observed between operations at 35 and 25 °C but further reducing temperature to 15 °C resulted in a decrease in rCOD from 91% to 86%.^65^

The impacts of other decision variables, such as single-stage vs two-stage configurations and with vs without effluent degassing, were also negligible in comparison (Figure S13,  < 0.02). This aligns with the finding from a previous TEA that two-stage anaerobic digestion (AD) has a higher methane yield than single-stage AD but requires greater capital investment and thus may not always be favorable.^54,66^ Several common features could be identified among the cheapest and the least carbon-intensive designs with different reactor types (Figure S14): they all operate at ambient temperatures without active H_2_ extraction, and they have HRTs of ≤4 days. Systems with the lowest LCs have a two-stage configuration without effluent degassing, but the single-stage alternatives with effluent degassing have significantly lower CIs with only slight increases in the LCs (Figure S14).

### Stage II. Key Driving Factors for System Sustainability

Results from the stage I analysis enabled us to narrow down the potential design space to the best-performing configurations: single-stage ambient-temperature systems with passive biogas collection and a short HRT (≤5 days). These designs were further examined through uncertainty and sensitivity analyses, with a bead size and bead lifetime varied within narrower ranges to exclude unlikely values based on published data.^7,16,55−57^ To have a more representative characterization of the performance and sustainability of the systems, a series of parameters in the process model was included to account for the technological uncertainties associated with different reactor types. Distributions of parameters varied in Monte Carlo simulations are detailed in Table S5.

#### COD Removal Performance under Uncertainty

**Figure 3:**
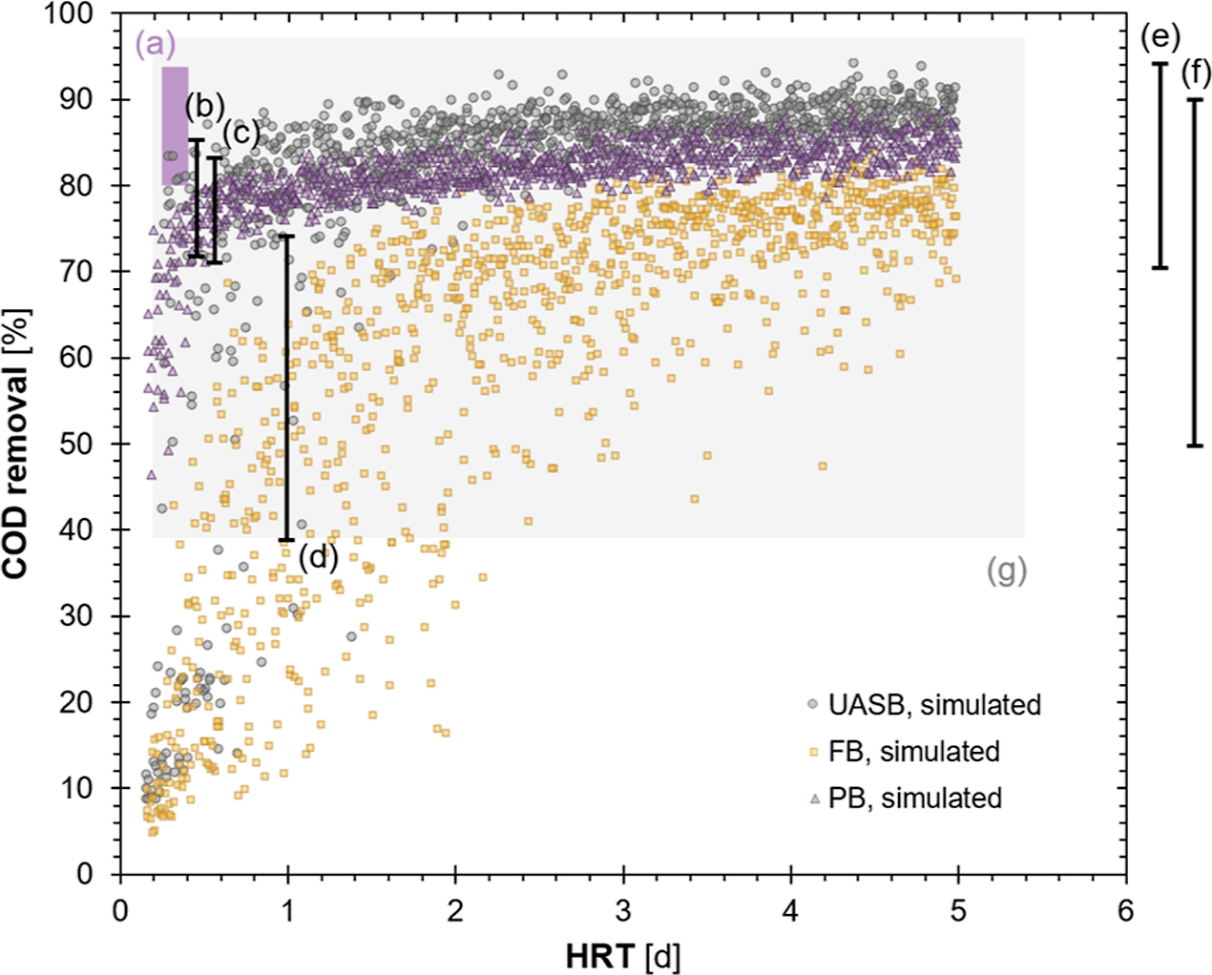
COD removal performance comparison between simulation results and lab- and full-scale data from the literature. FB—fluidized bed and PB—packed bed. (a) Bench-scale UASB and PB with biomass encapsulated with agar, calcium alginate, polyacrylamide, and poly(vinyl alcohol), synthetic wastewater;^14^ (b) full-scale UASB, brewery wastewater;^67^ (c) full-scale UASB, brewery wastewater;^68^ (d) full-scale UASB, brewery wastewater;^71^ (e) full-scale UASB, brewery wastewater, HRT not reported;^69^ (f) full-scale UASB, brewery wastewater, HRT not reported;^70^ and (g) full-scale UASB, multiple high-strength industrial wastewaters.^54^

Simulation results for packed bed systems demonstrated smaller predicted variances in steady-state rCOD than fluidized bed or suspended growth systems under uncertainty (Figure 3). The median (with fifth, 95th percentiles indicated in parentheses from hereon) rCODs were estimated at 86.0% (27.3–90.3%) for UASB systems, 69.3% (14.5–80.0%) for fluidized bed systems, and 82.1% (74.3–86.5%) for packed bed systems, suggesting that an encapsulated system with packed bed reactors could provide more reliable COD removal under varying conditions compared to the suspended growth systems. Furthermore, the simulated packed bed systems maintained over 46.5% COD removal under the least desirable conditions whereas UASB or fluidized bed could only achieve 4.8–8.5%. The simulated rCOD by UASB were generally consistent with full-scale performance data reported in the literature across a range of operating conditions and influent wastewaters (Figure 3b,c,e,f).^67−70^ Although experimental data in the literature were limited, a bench-scale study found packed bed reactors using four different hydrogels for encapsulation all reached over 80% rCOD within 20 days of continuous operation (Figure 3a). Although they demonstrated rCOD at or above simulated values, these experimental systems were operated at an elevated temperature (35 vs 22 °C in simulation) and a relatively low organic loading rate (2.9–4.8 vs 1.36–40.6 kg-COD·m^–3^·d^–1^ in this study), using methanol as the substrate.^14^

#### Life Cycle Cost and CI under Uncertainty

**Figure 4:**
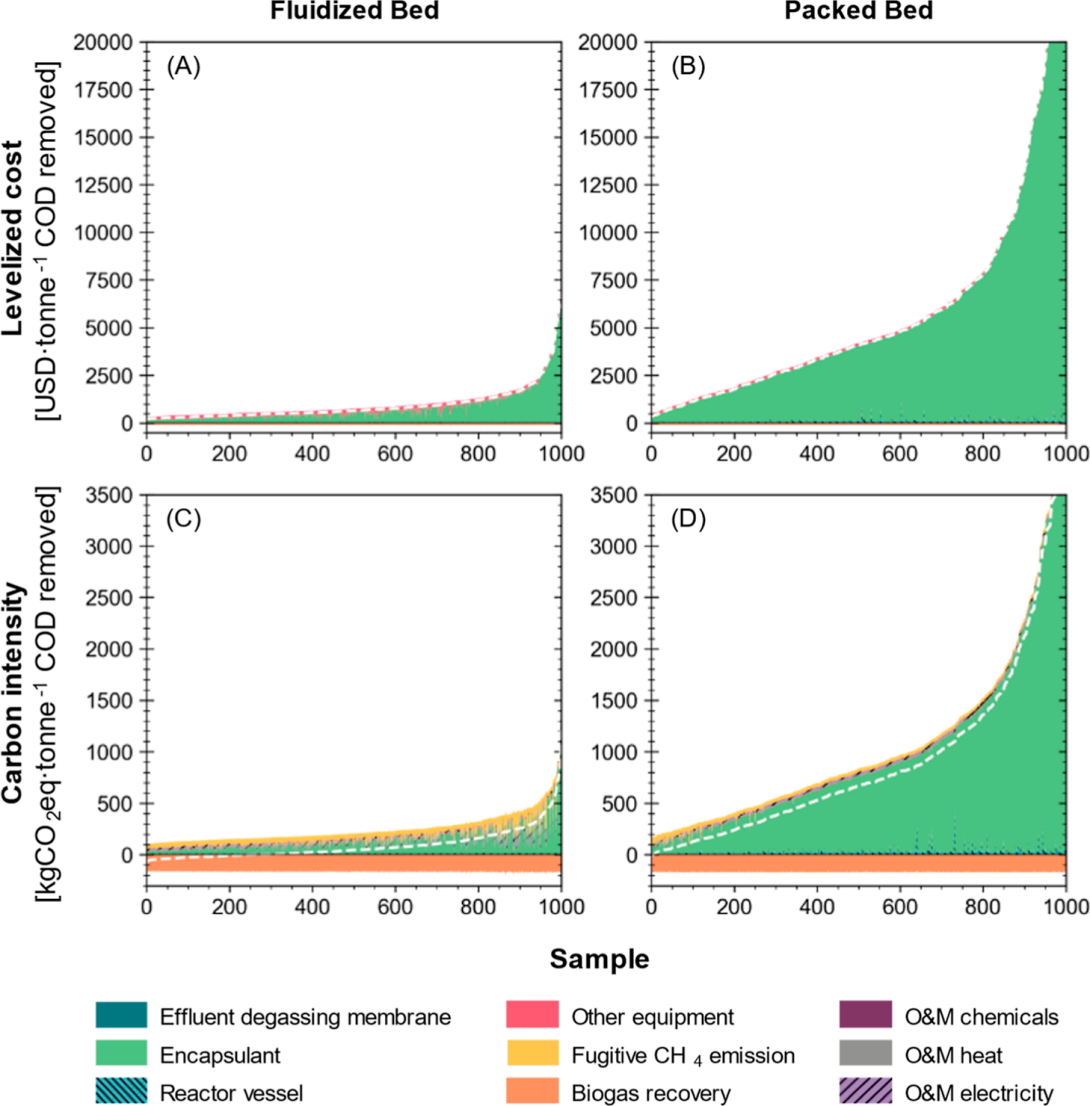
Breakdowns of the simulated (A,B) LC and (C,D) CI of COD removal by encapsulated systems using different types of reactors. Effluent degassing membrane is included in this figure to show its relative contribution to LC and CI compared to other items. The samples are sorted in ascending order of indicator values for better visualization and thus the *x*-axis value does not imply the actual order of simulation. White dashed lines indicate the net LC or CI of COD removal. LC and CI breakdowns of UASB systems can be found in Figure S15.

Packed beds tended to be the most expensive reactor type, with simulated LC of 57.9 (45.0–127) USD·tonne^–1^ COD for UASB (Figure S15), 655 (282–2507) USD·tonne^–1^ COD for fluidized bed (Figure 4A), and 4071 (776–18,989) USD·tonne^–1^ COD for packed bed systems (Figure 4B) with effluent degassing membranes. The LC of encapsulated systems strongly correlated with the amount of encapsulants used throughout the project lifetime, which accounted for 94.9% (79.8–98.9%) and 69.0% (33.9–91.6%) of the life cycle expenditure of packed bed and fluidized bed systems, respectively. A small-scale pilot study reported that alginate, unlike PEG, only accounts for 42% of the total initial material cost, but biomass washout and deterioration of COD removal caused by encapsulant disintegration were critical disadvantages to be overcome.^39^ The second largest contributor to the LC of packed bed systems (with a median contribution of 3.2%) was the capital investment for equipment (i.e., water pumps, iron sponge scrubber, double-membrane gas holder, and effluent degassing membrane contactor), which also accounted for significant shares of the life cycle expenditures for UASB (80.8%) and fluidized bed (26.3%) systems. When used for onsite heat generation as a substitute for natural gas, recovered biogas from an encapsulated anaerobic system can offset 13.7% (3.9–26.5%) or 2.5% (0.5–11.9%) of the life cycle costs, respectively, with fluidized bed and packed bed reactors.

Packed bed systems also had the highest estimated CI (672 [66.8–3008] kg CO_2_eq·tonne^–1^ COD removed) among reactor types with effluent degassing (Figure 4D). In comparison, the fluidized bed systems had a median CI of 45.9 (−37.8 to 363) kg of CO_2_eq·tonne^–1^ COD removed (Figure 4C). Embedded carbon emission in the encapsulant material was a dominant contributor to the CI of both encapsulated systems, accounting for 89.3% (55.8–97.3%) of packed bed systems’ and 50.1% (13.7–83.2%) of fluidized bed systems’ carbon emissions. Without biomass encapsulation, UASB systems had a median CI of −80.0 (−88.8 to 43.4) kg CO_2_eq·tonne^–1^ COD removed, with approximately 94% of the simulated samples being carbon negative. Similarly, a previous LCA study estimated an overall negative CI for a hypothetical anaerobic system treating industrial wastewater by recovering and reusing biogas in place of natural gas for the onsite steam boiler.^72^ However, the evaluated system had a much larger treatment capacity (2000 m^3^·d^–1^) and operation rather than construction was found to be the major contributor to negative environmental impacts, which is also seen in LCAs of anaerobic treatment of pulp and paper wastewater^73^ and brewery wastewater.^74^

Effective management of fugitive methane emission was considered essential for anaerobic treatment of industrial wastewater to have positive environmental benefits.^53^ Our simulations indicate that fugitive CH_4_ emissions contributed a considerable 29.8% (9.1–58.9%) to the total carbon emission of fluidized bed systems, even with effluent methane management. While eliminating the effluent membrane contactor had the potential to lower the LC to 616 (259–2491) USD·tonne^–1^ COD removed by lowering the required capital investment, the simulated increase in fugitive CH_4_ emission would outweigh the carbon savings from lower O&M electricity consumption and eventually drive the net CI up to 82.6 (−4.5 to 454) kg CO_2_eq·tonne^–1^ COD removed. The implications of effluent degasification on CI and LC were similar for the packed bed and UASB systems. This suggests that under current assumptions around membrane degasification technologies and performance there is a trade-off between LC and CI. More work is needed to explore alternative effluent methane management options both through experimentation and in an integrated analysis framework, with the goal of improving the synergy between economic and environmental sustainability of encapsulated anaerobic systems.

The recovered biogas was estimated to offset 203% (78.1–229%), 77.9% (31.7–131%), and 19.7% (5.1–71.2%) of the carbon emissions of systems with UASB, fluidized bed, and packed bed reactors, respectively, when displacing natural gas use at the brewery. The CI of recovered biogas consistently ranged between −205 and −149 kg of CO_2_eq·tonne^–1^ COD removed (Figure 4C,D) because the composition (i.e., the relative abundances of water vapor, CO_2_, H_2_, and CH_4_) and, thus, the lower heating values of recovered biogas had small variations across the simulated space of system design and operation. The recovered biogas could only offset 10% of operational carbon emissions in a previous study of decentralized treatment of gray and black wastewater with small-sized UASBs (0.6 m^3^·d^–1^) and adding an energy recovery system significantly raised the construction phase contribution to the life cycle environmental impacts.^75^

#### Key Design Decisions

**Figure 5:**
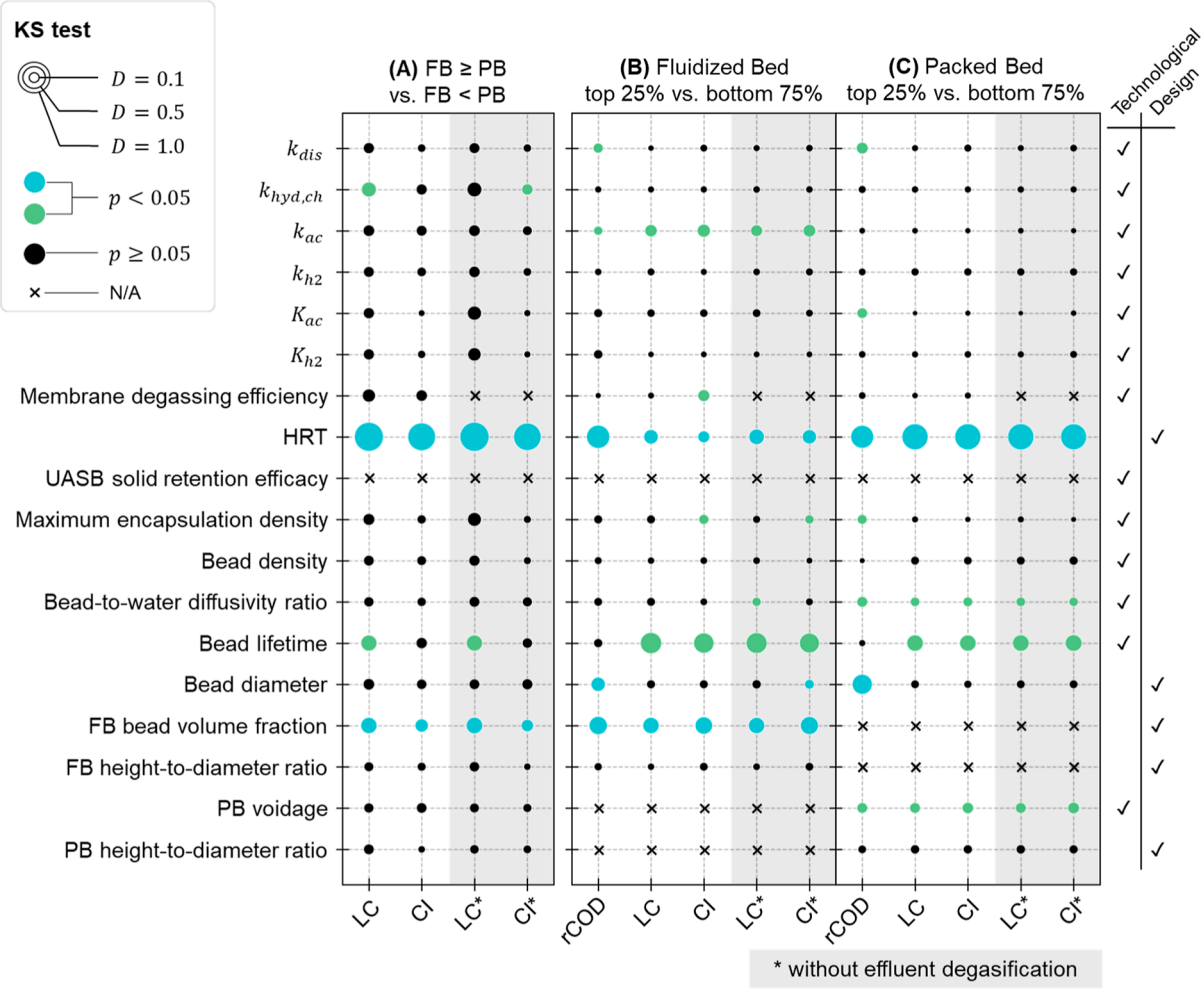
KS test results of parameter samples between two groups. Each bubble represents a single KS test on a parameter using one indicator as the grouping criterion. Bubble size indicates the value of the KS test statistics. A highlighted bubble indicates a statistically significant difference in parameter sample distributions between the two groups. Parameters were categorized based on whether they are technological uncertainty (highlighted in green if significant) or design decision variables (blue), which are also indicated by the √ marks in the right columns. FB—fluidized bed and PB—packed bed.

Through Monte Carlo filtering, the potential financial viability and environmental sustainability of early stage encapsulated anaerobic systems were found to be driven by a small number of decision variables and technological parameters. HRT was found to be the most important design decision to optimize in future R&D (Figure 5). rCOD was the most sensitive indicator to HRT for the fluidized bed systems (*D* = 0.63, *p* < 0.0001, Figure 5B), while LC (*D* = 0.79, *p* < 0.0001) and CI (*D* = 0.79 or 0.78, *p* < 0.0001) were more sensitive than rCOD (*D* = 0.61, *p* < 0.0001) for packed bed systems (Figure 5C). This finding stems from two key factors: (i) packed bed systems maintained over 74.3% COD removal for 95% of the simulated samples while the COD removal of fluidized bed systems was subject to a much higher uncertainty; and (ii) 55–65% of a packed bed reactor’s volume is filled with beads (compared to 3–25% for a fluidized bed reactor) and, as a result, the change in HRT leads to greater changes in the packed bed reactor size and the amount encapsulant material needed (the latter of which is the dominant contributor to its cost and impacts). Although a pairwise comparison showed a packed bed system always outperformed a fluidized bed system at rCOD under identical conditions, the significant sensitivity of LC and CI to HRT (*D* ∈ [0.87, 0.98], *p* < 0.0001; Figure 5A) suggests HRT is a key driver dictating whether a packed bed system would outperform its fluidized bed alternative. This means that the desirable range of HRT for fluidized bed designs likely differs from that for packed bed designs, which is further illustrated in stage III analysis.

Reducing bead diameter had a significant positive impact on rCOD for both reactor types (*D* = 0.24 or 0.47, *p* < 0.0001; Figure 5B,C) by increasing the specific interfacial surface area. It was also a significant driver for fluidized bed systems’ CI when effluent methane management was absent (*D* = 0.11, *p* < 0.05) because increasing bead diameter is expected to lead to greater O&M electricity required for fluidization. However, its impacts on other indicators were not significant relative to other technological uncertainty or decision decisions (*p* ∈ [0.06, 0.26]). For fluidized bed systems, all indicators were found to be sensitive to bead volume fraction in the reactor (*D* ∈ [0.31, 0.39], *p* < 0.0001) because this design decision, along with HRT, determines the total interfacial area and the total amount of encapsulant material in a reactor and they should be optimized simultaneously in the system design.

#### Driving Technological Parameters

Among sources of technological uncertainty, several encapsulant-related parameters stand out as important for future R&D. Bead lifetime did not affect rCOD but still had the greatest impacts on both encapsulated systems’ LC (*D* ∈ [0.32, 0.53], *p* < 0.0001) and CI (*D* ∈ [0.31, 0.48], *p* < 0.0001) (Table S5 and Figure 5B,C). All indicators of packed bed systems were sensitive to the uncertainty in bed voidage (*D* ∈ [0.13, 0.16], *p* < 0.01). Comparison of the distributions of packed bed voidage between the top 25% and the bottom 75% samples (Figure S16) suggested that the anticipated benefit of better COD removal from a lower voidage is unlikely to overcome the additional costs and impacts associated with more encapsulant materials required to make up a certain working bed volume. Therefore, it is recommended technology developers target loose and homogeneous packing throughout long-term operations of a packed bed system. Direction of the water flow and production of biogas may introduce more uncertainty to the bed voidage during operation and thus should be taken into consideration in system design. The uncertainty in substrate diffusivity through the encapsulation matrix (i.e., the bead-to-water diffusivity ratio) also had a significant impact on all packed bed indicators (*D* ∈ [0.10, 0.14], *p* < 0.05). CI of fluidized bed systems and rCOD of packed bed systems were also found mildly sensitive to the biomass encapsulation capacity (i.e., maximum encapsulation density). Given the importance of encapsulant materials to LC and CI, future R&D should prioritize the continued development of these materials as well as the characterization of correlations or interactions among different material properties to reduce the prediction uncertainty of system performance and facilitate sustainable design of the encapsulation matrix.

Among the ADM1 parameters, only *k*_ac_ (i.e., the maximum specific growth rate of acetoclastic methanogens; *D* ∈ [0.16, 0.20], *p* < 0.0001) was found to have significant impacts on fluidized bed systems’ LC and CI. In comparison, *K*_ac_ (i.e., the half saturation coefficient of acetate) had a significant impact on packed bed systems’ rCOD (*D* = 0.13, *p* < 0.01) but not on LC or CI. rCOD was also mildly sensitive to variations in *k*_dis_ (i.e., the first-order kinetic rate constant of particulate disintegration; *D* = 0.12 or 0.16, *p* < 0.01), but the effects were not strong enough to drive the LC or the CI of COD removal given uncertainty in other technological assumptions and design decisions. Nevertheless, these parameters should be prioritized for ADM1 calibration in future works to provide more accurate evaluations of the COD removal and methane production performance and to enable overall sustainability assessments of encapsulated anaerobic systems for similar applications.

### Stage III. R&D Priorities of the Encapsulated Anaerobic Technology

**Figure 6:**
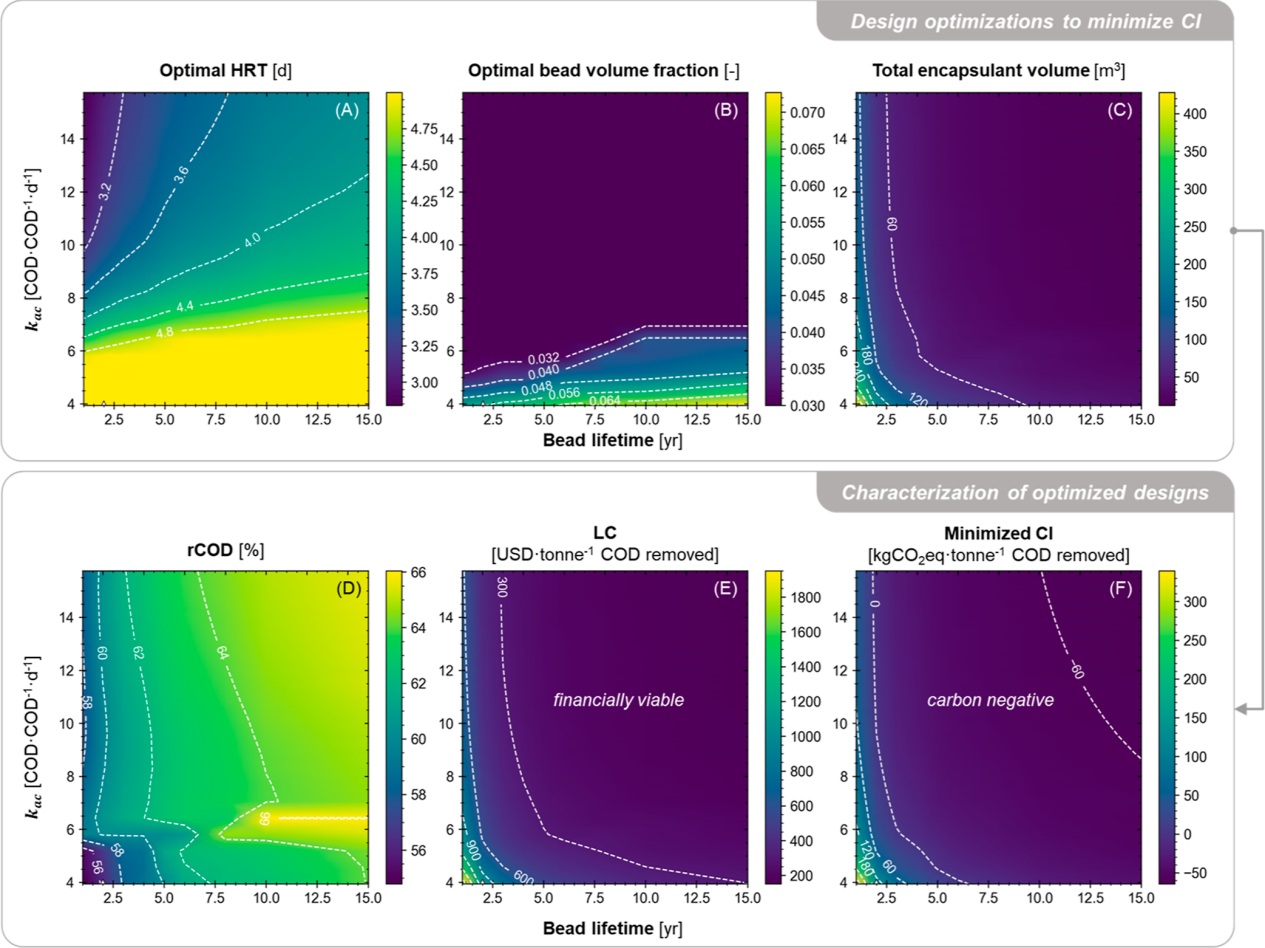
Mapping a fluidized bed system’s performance across ranges of bead lifetime and *k*_ac_ with 1 mm beads, while all other parameters are fixed at their baseline values. Colors and contour lines indicate (A–C) values of the design decision variables and (D–F) values of performance indicators with the tailored designs at given values of bead lifetime and *k*_ac_.

**Figure 7:**
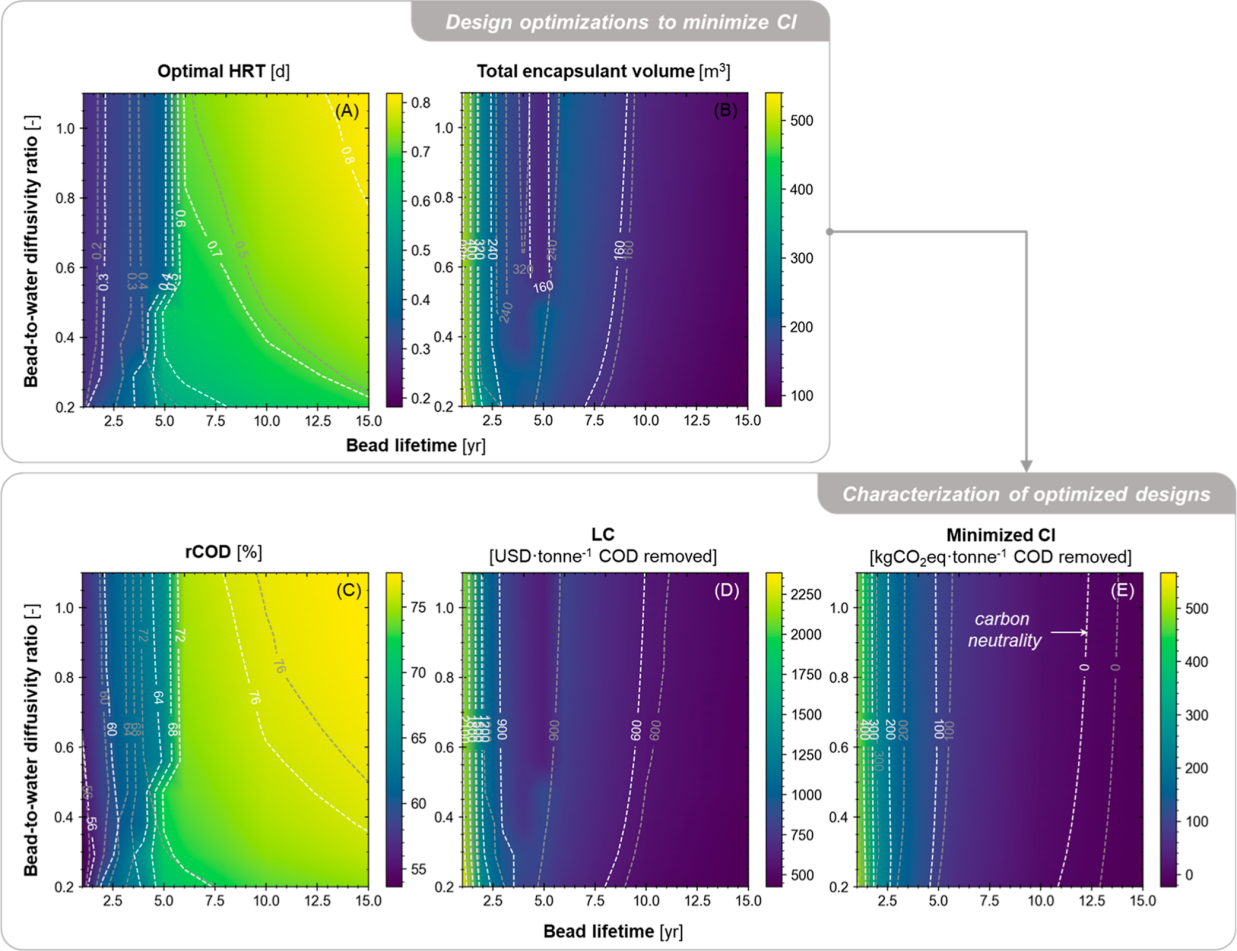
Mapping a packed bed system’s optimal performance across ranges of bead lifetime and bead-to-water diffusivity ratio with 1 mm beads while all other parameters are fixed. Colors and white contour lines indicate (A,B) tailored values of the design decision variables and (C–E) values of performance indicators with the tailored designs at given values of bead lifetime and bead-to-water diffusivity ratio, assuming loose packing of encapsulant beads (i.e., bed voidage = 0.45). Gray contour lines represent the dense packing scenario (i.e., voidage = 0.35) for comparison.

Fluidized bed and packed bed reactors were shown in stage I and stage II analyses to have their own advantages and disadvantages for small-scale applications of the encapsulated anaerobic technology. Monte Carlo filtering results suggest the sustainability of the two reactor types are likely to be driven by different sets of technological assumptions and design decisions. To delineate the sustainability frontier and to expedite technology R&D, the specific values of key design decision variables (i.e., HRT and bead volume fraction for fluidized beds, and HRT for packed beds; Figure 5B,C) that minimize CI were determined; this evaluation was performed across the two-dimensional space of the two most important technological parameters for each reactor type. Specifically, the HRT and bead volume fraction that yielded the lowest CI values for fluidized beds were determined across the uncertainty space (from reasonable minima to reasonable maxima) for the bead lifetime and *k*_ac_ (Figure 6), and the HRT that yielded the lowest CI for packed beds was determined across the uncertainty space for the bead lifetime and bead-to-water diffusivity ratio (Figure 7). HRT was bounded between 1 h and 5 days and fluidized-bed bead volume fraction was constrained between 0.03 and 0.25. To maximize specific interfacial area for mass transfer, both systems were assumed to use 1 mm beads, which is the lower bound for bead sizes seen in wastewater-related applications in the literature.^16,55−57^ For packed bed systems, loose packing (i.e, voidage = 0.45) was assumed.

The minimum potential CI of these tailored designs was estimated to be between −64.5 and 339 kg of CO_2_eq·tonne^–1^ COD removed for fluidized bed designs (Figure 6F) and between −22.9 and 510 kg of CO_2_eq·tonne^–1^ COD removed for packed bed designs (Figure 7E). In comparison, centralized WRRFs (>10,000 population equivalent) using a conventional activated sludge process have been estimated to consume 0.79–1.07 kW h electricity per kg COD removed on average,^76^ which translates to a CI of 348–471 kg CO_2_eq·tonne^–1^ COD removed under identical assumptions of grid electricity CI. Additionally, onsite fugitive emissions of CH_4_ from the centralized WRRFs (using aerobic treatment) account for another (roughly) 210 kg CO_2_eq·tonne^–1^ COD removed.^77^ This suggests that encapsulated anaerobic systems with design optimization have the potential to consistently provide distributed COD removal at a lower CI than the average centralized WRRFs. Moreover, with improvements in critical technological parameters, both systems could potentially be deployed and operated with a negative CI at small- or medium-sized industries where more traditional technologies, such as UASBs, might be infeasible.^54^ The LCs were estimated to be 151–1950 USD·tonne^–1^ COD removed with fluidized beds and 426–2329 USD·tonne^–1^ COD removed with packed beds. The low values within these ranges are similar to or less than charges incurred by discharging to a centralized WRRF (e.g., 322–1340 USD·tonne^–1^ COD discharged^78,79^). This means for small- or medium-sized industries, onsite deployment of this technology also has a chance be financially more desirable than directly discharging high-strength wastewater to a centralized WRRF.

For fluidized bed systems, a critical R&D pathway toward sustainable distributed treatment is to simultaneously improve encapsulant longevity and the bioreactivity of encapsulated acetoclastic methanogens (Figure 6E,F). When both bead lifetime is short (e.g., <5 years) and *k*_ac_ is small (e.g., <6 COD COD^–1^ d^–1^), increasing either parameter without compromising the other can lead to significant reductions in CI and LC. The latter could be achieved through optimization of the microbial community prior to encapsulation and/or control of the encapsulant internal environment.^80^ The tailored bead volume fraction (to minimize CI) is generally small (3.0–6.0% of bed volume, Figure 6B), but a longer HRT (>4.8 d, Figure 6A) will likely be needed to maintain a significant COD removal (55–61%) in this region. The tailored encapsulant volume in a fluidized bed reactor generally decreases with *k*_ac_ and increases with the bead lifetime. Beyond this region, further improvement of a single parameter (either bead lifetime or *k*_ac_), while the other remains weak has diminishing marginal benefits in cost or CI reduction. Although further increasing *k*_ac_ will enable a similar COD removal with a smaller reactor or less beads, the total amount of encapsulant material required for the 30 yr project lifetime barely decreases because frequent bead replacements are needed for the short bead lifetime (Figure 6C). Similarly, the tailored HRT or bead volume fraction cannot afford to be too low if *k*_ac_ remains small, which limits the benefits that can be gained from fewer bead replacements by further improving bead longevity to 6, 8, 10, and again 15 years.

Compared to fluidized beds, R&D of packed bed systems should prioritize increasing bead longevity over any other technological parameters because it dictates the frontier of system sustainability (Figure 7D,E). Both LC and CI can be significantly reduced by increasing the bead lifetime from 1 to 3 years. A longer bead lifetime also allows the system to target a higher rCOD by designing a larger packed bed reactor (i.e., optimal HRT increases from approximately 6.5 to 14 h, Figure 7A). Although PEG hydrogel had been estimated to have a lifetime over 10 years in the literature,^7^ it was found in preliminary experiments that the addition of microbial cells and mixing high strength wastewater in the reactor could affect the structural integrity of the beads and significantly reduce lifetime to as short as 30 days.^43^ If the goal of the system is carbon neutrality (i.e., CI = 0 kg of CO_2_eq·tonne^–1^ COD removed), using a packed bed reactor would require the beads to last at least 11 years without replacement, but the required bead longevity can be as short as 2 years with fluidized beds. A lower bed voidage (i.e., gray contour lines in Figure 7) would make it even more difficult to achieve carbon neutrality. Unlike fluidized bed systems, increasing diffusivity through the encapsulation matrix within the evaluated range has minimal impact on the optimal sustainability of packed bed systems because the negative effect of low diffusivity on COD removal may be largely overcome by design decisions.

Despite the higher LC and CI, packed bed reactors may be preferred over fluidized beds due to locality-specific contextual factors. If the industry is bound by a discharge permit but has a limited physical space, then an optimized packed bed design may make it possible for the system to consistently achieve higher COD removal than if a fluidized bed of the same size is used. Depending on bead longevity, packed bed systems with a 50 m^3^·d^–1^ treatment capacity would have a tailored reactor size between 33 and 100 m^3^, a much smaller footprint than fluidized bed designs (i.e., 161–297 m^3^) for similar levels of COD removal. This is mainly attributed to the difference in the optimal HRT between the reactor types from 6.5 to 20 h for packed beds compared to 2.8–5.0 days for fluidized beds.

The most critical R&D pathway for encapsulated systems also depends on contextual factors. For example, this study assumed the system would be deployed at a medium-size brewery that purchases natural gas for heating onsite. If affordable low-CI energy for heating is available, the R&D priorities could shift away from optimization of the methanogenic microbial community at room temperature, because anaerobic bioreactivity can often gain significant improvement by operating the system at mesophilic temperatures.^58^ Although not explicitly captured by the model, tensions may exist between different properties of encapsulant materials, which could limit the feasible region for technological advancements in Figures 6 and 7. Additionally, the environmental implications of end-of-life disposal of encapsulant materials are currently highly uncertain but could play a significant role in the overall sustainability of the technology. Improving encapsulant longevity could make the beads less biodegradable and could have unintended consequences (e.g., to human health or biodiversity) if they are released into the environment without control. Strategies for reuse, recycling, or safe disposal of the beads should be developed in conjunction with improvements in the durability of encapsulant materials. Unequivocally, lowering the cost and impacts associated with the use of encapsulant materials is critical for the overall sustainability of this technology, as well as broader applications of biomass encapsulation regardless of design or other technological assumptions.

Moving forward, more work is needed to systematically evaluate the implications of different material choices or technological advancements. Knowledge and data from experiments should be consolidated to establish quantitative connections among encapsulant material properties (biocompatibility, durability, degradability, density, etc.) and empirically outline the feasible region of key technological parameters (e.g., bead lifetime, diffusivity, and encapsulation capacity). Rigorous calibration and validation of the multiscale process model with experimental data across diverse conditions (e.g., operating pH, influent wastewater types) will also enable better performance prediction and more specific recommendations for optimal system design.

## Supporting Information Available

Biochemical process model, mass-transfer models, and verification against batch experimental data, system design algorithms for generating life cycle inventories, detailed TEA and LCA assumptions and data, assumptions and parameter values for stage I and stage II analyses, relative impacts of additional discrete design decisions, correlations between simulated LC and CI, breakdowns of life cycle cost and environmental impacts of the best configurations in stage I analysis, breakdowns of LC and LC of UASB systems in stage II analysis, and additional Monte Carlo filtering results (PDF)
