# Supplementary material for: Prioritization of Early-Stage Research and Development of a Hydrogel-Encapsulated Anaerobic Technology for Distributed Treatment of High Strength Organic Wastewater
Source: Environ Sci Technol. 2024 Oct 26;58(44):19651–65. doi: 10.1021/acs.est.4c05389 (PMC11542886; doi:10.1021/acs.est.4c05389)
Supplement: Supplementary file 1 — es4c05389_si_001.pdf [file es4c05389_si_001.pdf]

Supporting Information for

Prioritization of early-stage research and development of a hydrogel-encapsulated anaerobic technology for distributed treatment of high strength organic wastewater

Xinyi Zhang[1], William A. Arnold[2], Natasha Wright[3], Paige J. Novak[2], Jeremy S. Guest[1,4,5]*

[1] Department of Civil and Environmental Engineering, University of Illinois Urbana-Champaign, 3221 Newmark Civil Engineering Laboratory, 205 N. Mathews Avenue, Urbana, IL 61801, USA.

[2] Department of Civil, Environmental, and Geo- Engineering, University of Minnesota, 500 Pillsbury Drive S.E., Minneapolis, MN 55455, USA.

[3] Department of Mechanical Engineering, University of Minnesota, 111 Church Street SE, Minneapolis, MN 55455, USA.

[4] Institute for Sustainability, Energy, and Environment, University of Illinois Urbana-Champaign, 1101 W. Peabody Drive, Urbana, IL 61801, USA.

[5] DOE Center for Advanced Bioenergy and Bioproducts Innovation, University of Illinois Urbana-Champaign, 1206 W. Gregory Drive, Urbana, IL 61801, USA.

*Corresponding author: jsguest@illinois.edu, +1 (217) 244-9247

33 pages, 7 tables, 16 figures

Table of Contents

S1. Process model ... S2
S2. System design ... S13
S3. Techno-economic analysis (TEA) and Life cycle assessment (LCA) ... S17
S4. Discrete design decision analysis settings ... S21
S5. Monte Carlo simulation settings ... S23
S6. Additional results ... S24
References ... S29

S1. Process model

We developed a multi-scale model to provide a pseudo-mechanistic description of the biological and physio-chemical processes in the encapsulated anaerobic reactors (**Figure S1**). At the encapsulation matrix scale, special emphasis was placed on describing the mass transport characteristics of components of different particle sizes (e.g., particulate substrates like lipids vs. soluble substrates like volatile fatty acids [VFAs]). At the reactor scale, changes in bulk liquid flow patterns stemming from specific reactor designs were characterized. Biochemical reactions were considered to take place both in the bulk liquid and within the encapsulation matrix.

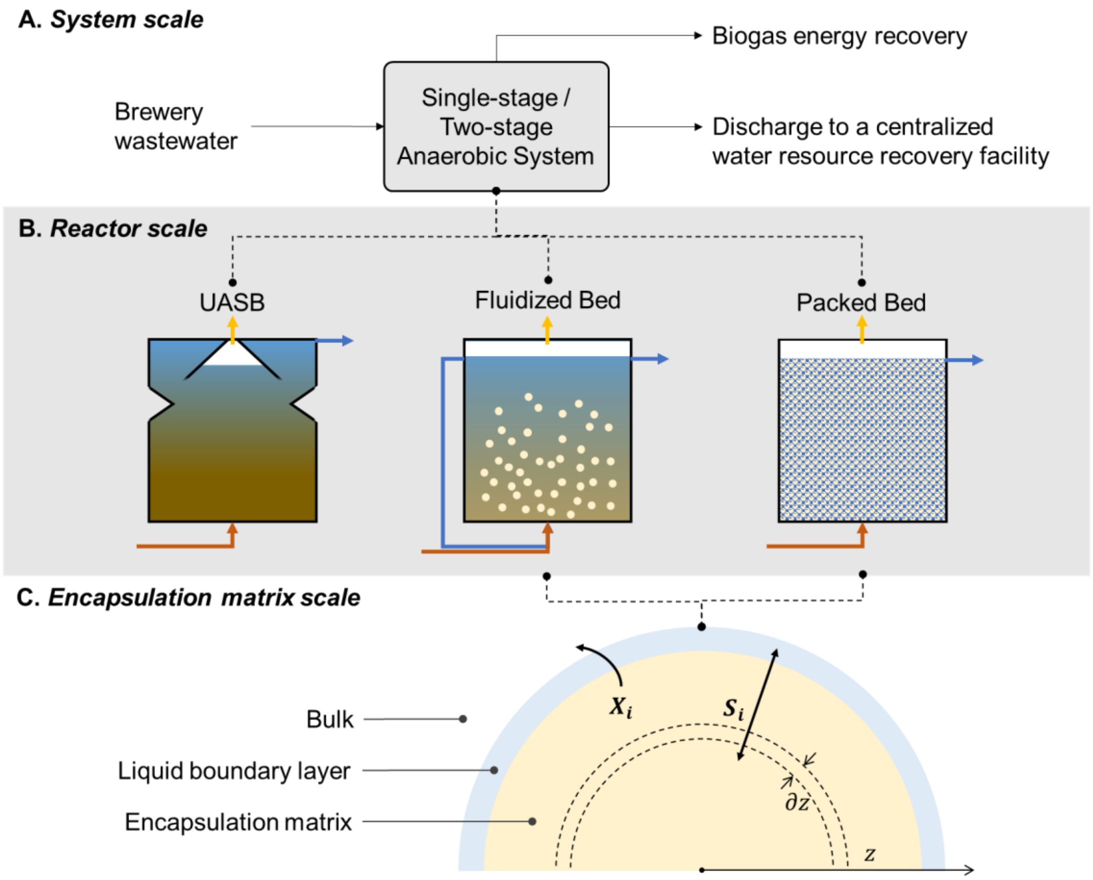

Figure S1. A simplified diagram of the encapsulated anaerobic process model. UASB – upflow anaerobic sludge blanket; ∂z – control volume; S_i – a soluble component, mass balance governed by diffusion and reaction; X_i – a particulate component, mass balance governed by detachment and reaction.

S1.1 Biochemical model

The anaerobic biochemical reactions in the bulk liquid and encapsulation matrix are both represented by the stoichiometry and kinetics of the Anaerobic Digestion Model no.1 (ADM1).[1] We adapted the pH model in ADM1 to enable estimation of the acid/base addition required to control bulk pH. We then fractionated the influent brewery wastewater across ADM1 state

variables to yield 6,800 mg·L^{-1} total COD and 5,620 mg·L^{-1} soluble COD, 3,000 mg COD·L^{-1} of which was in form of monosaccharides and 1,200 mg COD·L^{-1} of which was represented as VFAs (**Table S3**).

To verify the computational implementation of ADM1 in QSDsan, a single-stage CSTR was modeled in QSDsan and simulated with 100 different initial conditions, which were randomly sampled from uniform distributions (±50%) around the initial state used in an IWA MATLAB/Simulink implementation.[2] All ADM1 parameters and assumptions regarding the CSTR were set to be identical to the ADM1 implementation within the BSM2 framework.[2,3] The CSTR was able to reach steady states within the simulated time span (200 days) regardless of initial conditions. The steady-state values of all ADM1 state variables simulated with QSDsan were compared against the reported values and showed good alignment (**Figure S2-Figure S4**).

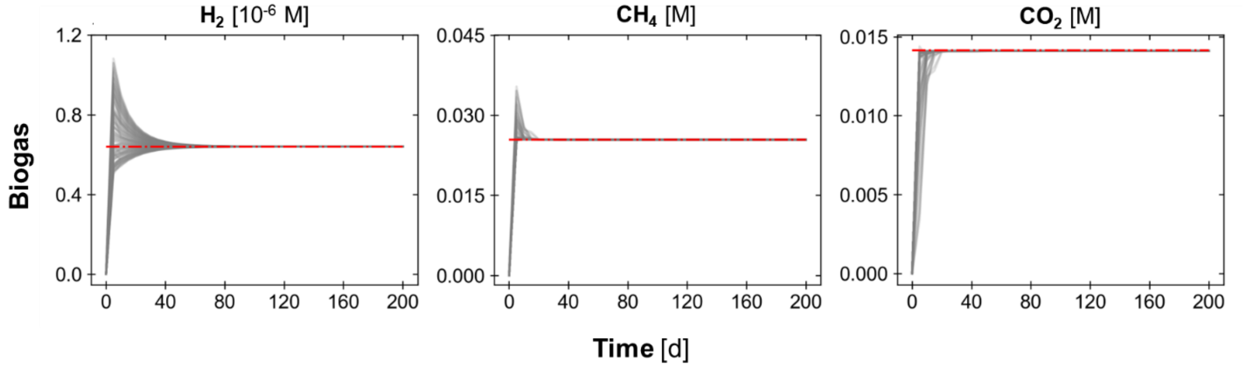

Figure S2. Dynamics of ADM1 biogas state variables in the headspace of an anaerobic CSTR simulated with QSDsan (with 100 different initial conditions) and reported values with identical simulation settings. Each grey solid line represents one simulation with a distinct initial condition. Red dash-dot lines indicate the reported steady-state values.

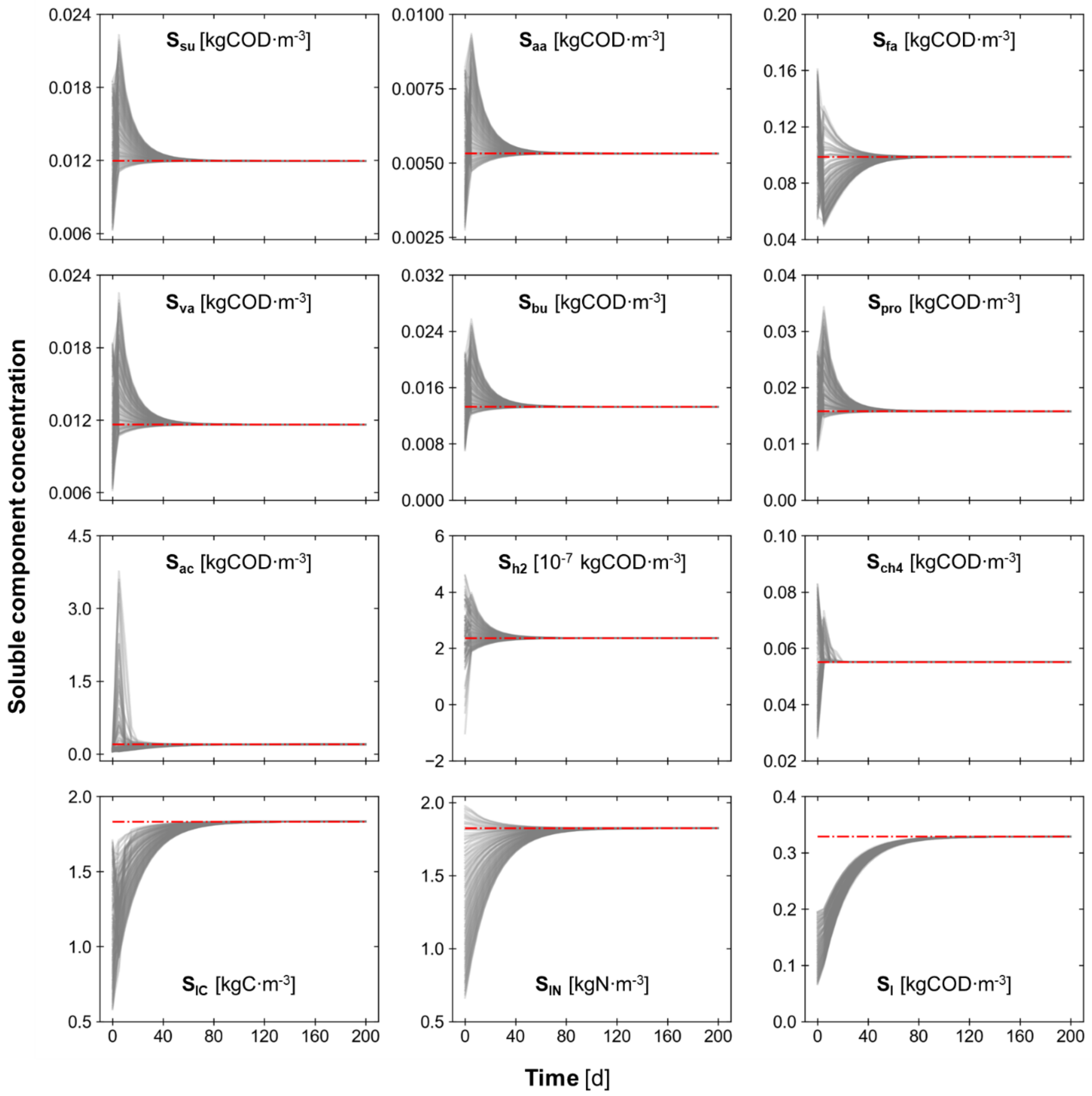

Figure S3. Dynamics of ADM1 soluble state variables in an anaerobic CSTR simulated with QSDsan (with 100 different initial conditions) and reported values with identical simulation settings. Each grey solid line represents one simulation with a distinct initial condition. Red dash-dot lines indicate the reported steady-state values.

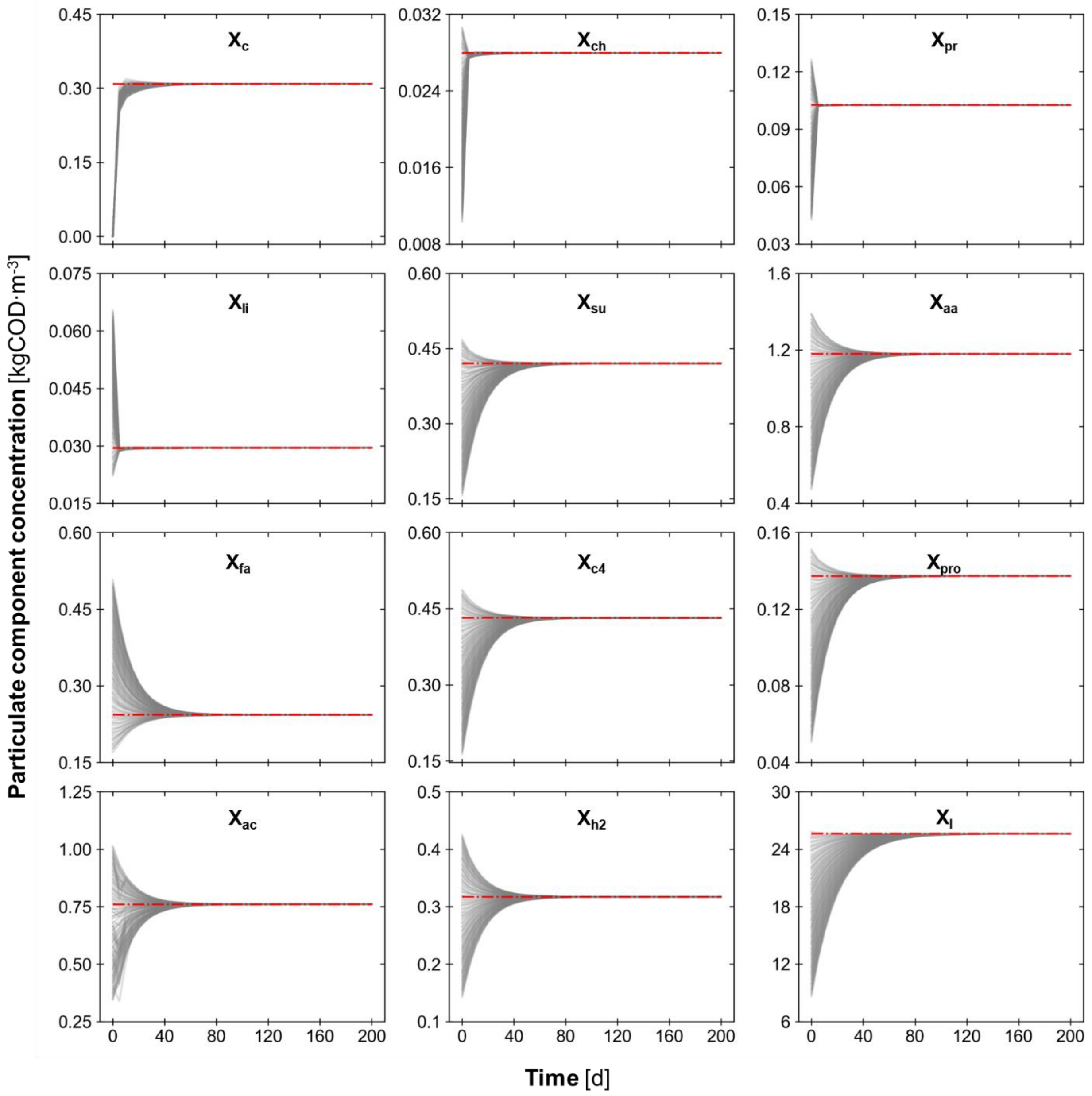

Figure S4. Dynamics of ADM1 particulate state variables in an anaerobic CSTR simulated with QSDsan (with 100 different initial conditions) and reported values with identical simulation settings. Each grey solid line represents one simulation with a distinct initial condition. Red dash-dot lines indicate the reported steady-state values.

S1.2 Mass transfer models

S1.2.1 Micro-scale mass balance

At the encapsulation matrix scale (**Figure S1C**), we constructed a one-dimensional model to describe the mass transfer of the soluble (S) and particulate (X) components in ADM1 through the spherical encapsulant beads and the liquid boundary layer around them. For soluble components (S_i), the mass balance is primarily comprised of two terms – diffusion as described by Fick's Law and transformation as described by ADM1 (**Eq. S1**):

$$\frac{\partial S_i}{\partial t} = D_i\left(\frac{\partial^2 S_i}{\partial z^2} + \frac{2}{z}\frac{\partial S_i}{\partial z}\right) + R_{S_i} \tag{S1}$$

where S_i is the soluble component concentration at location z, which describes the radial distance from the core of a spherical bead. D_i represents the diffusivity of component S_i in bulk liquid or through the encapsulation matrix. The diffusivity through encapsulant was assumed to be a fraction of that in pure water. R_{S_i} represents component S_i's local rate of transformation. Liquid-gas transfer was considered only in bulk. The boundary conditions for soluble components include (i) no diffusive flux at the core of the beads (i.e., at $z = 0$) and (ii) concentration continuity at the bead-liquid boundary layer (LBL) interface (i.e., at $z = L_F$).

For particulate components (X_i), the mass balance in the encapsulation matrix is assumed to be governed by two mechanisms – detachment and transformation (including growth, decay, disintegration, and hydrolysis). Once detached, particulate components cannot re-attach to the encapsulant but instead enter the bulk liquid. The mass balance is described by **Eq. S2**:

$$\frac{\partial X_i}{\partial t} = R_{X_i} - R_{de,X_i} \tag{S2}$$

where R_{de,X_i} is the local rate of detachment of component X_i. In contrast to biofilms, it is assumed that encapsulant beads maintain an unchanged shape and size throughout their lifetime. The rate of detachment at any location in the encapsulation matrix increases with the local concentration of particulate components so that the encapsulated particulates do not exceed a maximum encapsulation density [g-TSS·(L-beads)$^{-1}$] (**Eqs. S3-S6**):

$$R_{de,X_i} = f_{TSS} \cdot k_X^{net} \cdot X_i \tag{S3}$$

$$f_{TSS} = \frac{1}{1 + \exp(K_{TSS} - TSS)} \tag{S4}$$

$$k_X^{net} = \frac{\sum_i R_{X_i}}{\sum_i X_i} \tag{S5}$$

$$TSS = \sum_i X_i f_{SS,X_i} \tag{S6}$$

where k_X^{net} is the net production rate constant of all particulates. f_{TSS} is a scaling factor evaluated between 0 and 1 depending on the values of TSS (the total mass concentration of particulate components, in kg solids·m^{-3} beads) and K_{TSS} (the half-saturation coefficient of particulate detachment). K_{TSS} is thus defined as ½ of the maximum encapsulation density of the beads. f_{SS,X_i} is the unit conversion factor for component X_i, e.g., in kg TSS · kg^{-1} COD for biomass components. The average detachment rate of X_i per unit volume of beads (i.e., the rate of mass transfer to bulk) can be derived from a spatial integration (**Eq. S7**):

$$\bar{R}_{de} = \frac{\int \sum_i R_{de,X_i}(z)\, dV}{\int dV} \tag{S7}$$

The mathematical form of this model was chosen such that i) the rate of detachment of a certain component is proportional to its relative abundance among all particulate components at the location; ii) the overall rate of solid detachment approaches the net solid production rate (derived from local biochemical reactions) as the local solid concentration approaches the maximum encapsulation density; iii) when the encapsulant is not "saturated" with particulates, the rate of solid detachment is a fraction of the net production rate; iv) the fraction stays close to zero until the local solid concentration approaches the half saturation level (**Figure S5**); and v) the detachment rate is smooth with respect to the local concentrations of particulate components.

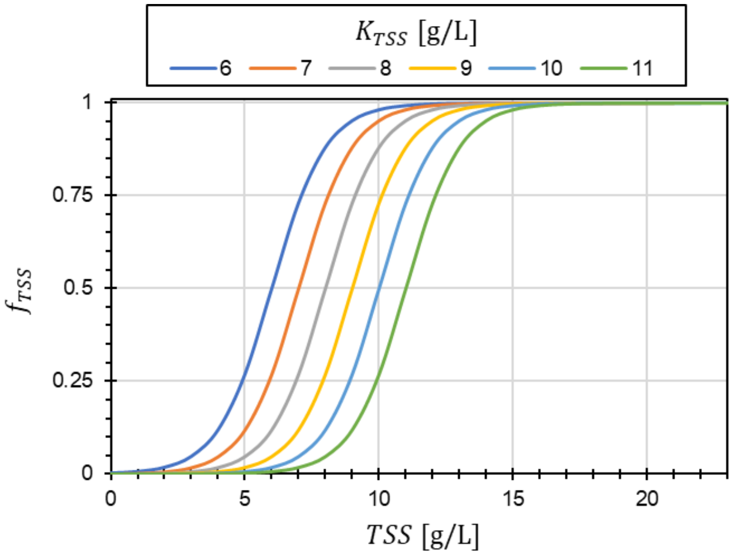

Figure S5. Illustration of the relation between local solid concentration and the scaling factor of solid detachment rate given different maximum encapsulation densities.

The resulting system of partial differential equations (PDEs) was solved by discretizing the spatial dimension using the method of lines[4] and approximating the spatial derivatives with centered differences.

S1.2.2 Micro-scale mass transfer model verification

To verify the micro-scale mass transfer model, a series of convergence tests were performed. First, an anaerobic CSTR with encapsulated biomass was simulated with varying bead diameters (10mm, 2mm, 1mm, 0.2mm, 0.02mm) and HRTs (24h, 12h, 10h, 8h, 4h, 2h, 1h) till it reached steady states. It was observed that for a given bead size, steady-state COD removal percentage consistently increased with increasing HRT (**Figure S6**). For a given HRT, COD removal mostly decreased consistently with increasing bead size, with the exception that with short HRT, moderate bead size (1-2 mm diameter) performed better than large or small bead sizes.

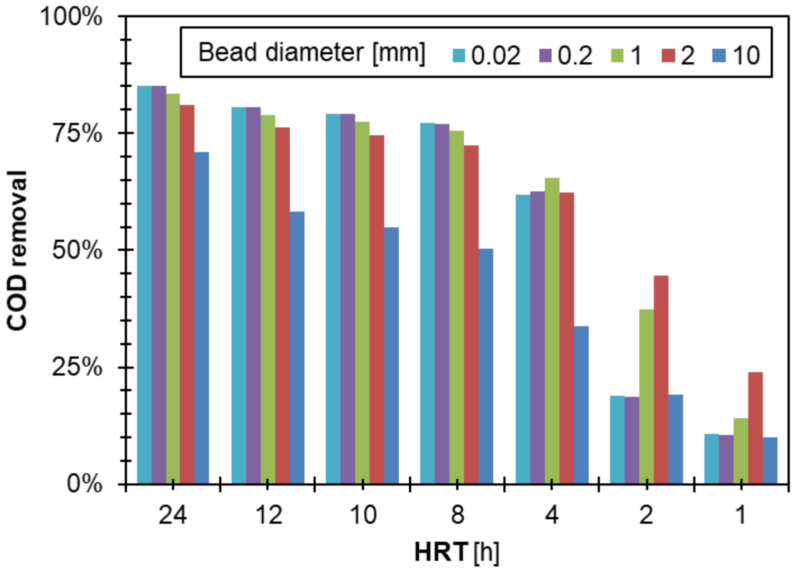

Figure S6. Simulated steady-state COD removal by an encapsulated anaerobic CSTR with varying HRT and bead size.

With a small bead size (20 μm diameter), the encapsulated CSTR was expected to behave similarly to the CSTR with suspended growth biomass when all other conditions are kept identical. To verify this presumption, the encapsulated CSTR was first simulated with various bead volume fraction (10-59%) and HRT (1-24h). After each simulation, the solid residence time (SRT) of the encapsulated biomass was derived from the steady-state data and the solid retention efficacy (f_{retain}) of a CSTR with suspended growth biomass was adjusted to match the SRT exactly using the following equations.

$$SRT = \frac{V \cdot X_{biomass}}{Q \cdot X_{biomass}^{e}}$$

$$\tau = \frac{V}{Q}$$

$$X_{biomass}^{e} = (1 - f_{retain}) \cdot X_{biomass}$$

$$\therefore f_{retain} = 1 - \frac{\tau}{SRT}$$

The COD removal and the overall biomass TSS concentration for both reactors were then estimated for comparison (**Figure S7**). Results showed good convergence of COD removals between the two reactors, as indicated by data points along the diagonal dashed line in **Fig. S7**. The mean absolute pair-wise difference in COD removal was 2.6%. All data points located above the diagonal line, indicating the suspended growth CSTR had slightly better COD removal than the encapsulated CSTR.

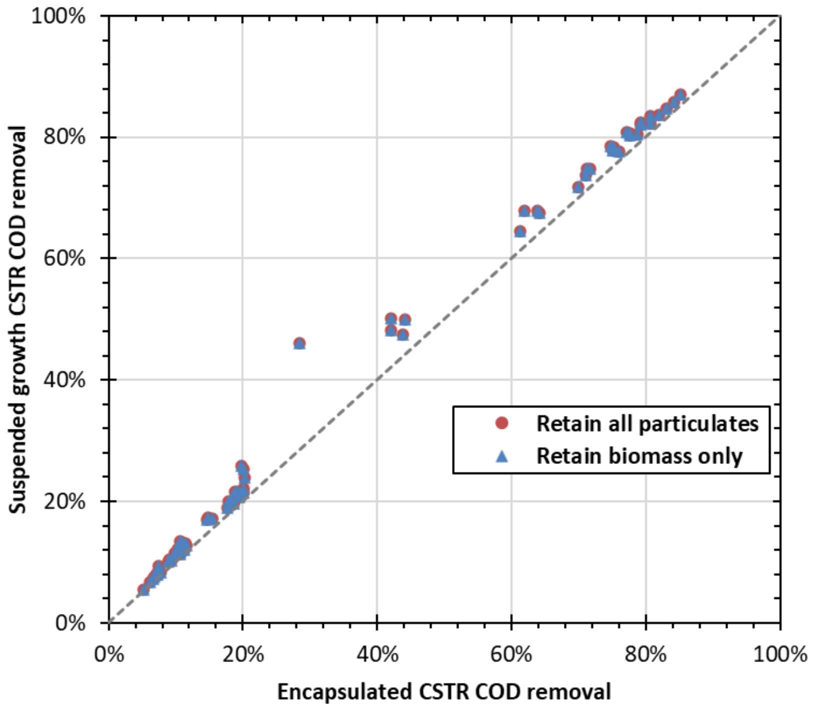

Figure S7. Pair-wise comparison of simulated steady-state COD removals between encapsulated and suspended growth CSTRs of identical SRTs and HRTs. Red dots indicate the suspended growth CSTR retains all particulate components indifferently. Blue triangles indicate only biomass components were subjected to ideal retention. Real-life reactors, such as UASBs, should behave between these two scenarios.

Finally, the encapsulated CSTR was simulated with different resolutions for spatial discretization (n = 5, 10, 20). Similar spatial distributions of ADM1 component concentrations were observed across all resolutions (**Figure S8**). The maximum relative error of steady-state COD removal [%] between high and low resolutions was below 0.11%. Therefore, n = 5 was deemed sufficient for simulating the effect of mass transfer for encapsulated anaerobic COD removal. In all simulations, n = 10 if the bead diameter was greater than 5 mm, and n = 5 otherwise.

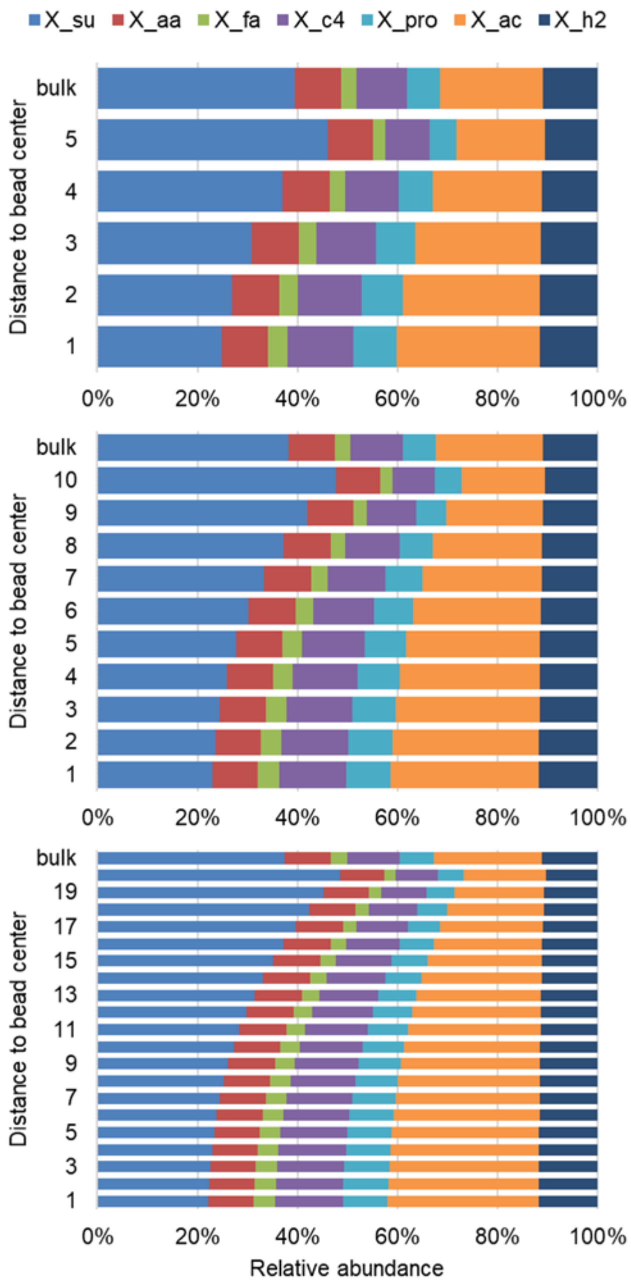

Figure S8. Relative abundance of biomass components in different discretized locations within the encapsulation matrix. Top, middle, and bottom panels correspond to discretizing the encapsulant bead into 5, 10, and 20 layers, respectively, along its radius.

S1.2.3 Reactor-scale mass balance

From preliminary experiments and economic analysis, mechanical mixing was found to be energy-intensive and detrimental to encapsulant bead integrity.[5] Therefore, two reactor types – a fluidized bed reactor with a fine mesh to retain the beads and a packed bed reactor – were

considered as potential designs for the encapsulated anaerobic system (**Figure S1B**). For comparison purposes, a UASB reactor was also modeled in this study.

Consistent with the GPS-X simulation software,[6] we modeled the UASB reactor as a continuous stirred tank reactor (CSTR), in which the HRT was defined with respect to the total liquid phase volume (i.e., including the sludge volume). The substrate diffusion into the sludge granules was assumed to not be limiting and thus the biochemical processes are modeled like suspended growth systems. A fixed solid retention efficacy was assumed for the phase separator for simplicity (**Table S5**).

A single CSTR was also used to describe the hydrodynamics of the fluidized bed reactor. External recirculation was applied to promote mixing and fluidize the encapsulation beads. The minimum fluidizing velocity, and thus the minimum recirculation ratio, was estimated by solving the Ergun equation with given properties of beads and bulk liquid as well as an assumed void fraction of the bed prior to fluidization (**Section S2.4**).[7] The packed bed reactor was modeled as multiple CSTRs in series or as a single CSTR when external recirculation was applied (**Section S2.1**). A common gas phase and a unified volumetric mass-transfer coefficient ($k_L a$) value was assumed for all CSTRs. For both fluidized bed and packed bed reactors, the HRT was defined with respect to the bulk liquid phase only, excluding the bead volume. The mass balance for the bulk liquid in a single CSTR can be described as follows (**Eqs. S8-S9**):

$$\frac{dS_{B,i}}{dt} = \frac{Q\left(S_{in,i} - S_{B,i}\right)}{V_B} - \frac{A_F}{V_B}\, k_{BL,i}\left(S_{B,i} - S_{L_F,i}\right) + R_{S_{B,i}} \tag{S8}$$

$$\frac{dX_{B,i}}{dt} = \frac{Q\left(X_{in,i} - X_B\right)}{V_B} + R_{X_{B,i}} + \bar{R}_{de,i} \cdot \frac{V_{beads}}{V_B} \tag{S9}$$

where subscripts in, B, and L_F indicate influent, bulk, and the bead-LBL interface, respectively. $k_{BL,i}$ indicates the resistance of mass transfer through the LBL. $k_{BL,i}$ was determined by substrate diffusivity in water and LBL thickness. We assumed identical $k_{BL,i}$ values between fluidized bed and packed bed reactors because preliminary simulations suggested assumptions of LBL thickness had negligible impacts on system effluent COD at steady state (< 0.2% absolute difference). V_{beads} and A_F represent the total volume and the total interfacial area of beads, respectively, in the CSTR. Q is the bulk liquid forward flowrate.

S1.3 Verification against batch experimental data

Bench-scale experiments have been conducted to investigate the biocompatibility and durability of PEG beads.[8] Anaerobic digester sludge (ADS) from a wastewater treatment plant was used to prepare three distinct types of PEG beads for the experiments: beads encapsulating concentrated ADS as received (which yields approximately 180 mg-protein·L^{-1} biomass), anaerobic granules, or powdered activated carbon (PAC)-supported biofilms formed using synthetic wastewater and the ADS. The synthetic wastewater was prepared following Zhu et al.'s recipe.[9] Triplicate serum bottles sealed and attached with 5-mL glass syringes were used as batch reactors, each

containing 25 mL synthetic wastewater and approximately 10 mL PEG beads. The batch reactors were constantly agitated, and the reaction temperature was held at 37°C. Methane production was measured daily for 7-10 days.

To verify the process model, a complete mix batch reactor was simulated for 8 days using baseline values for ADM1 kinetic and stoichiometric parameters. Volumes of liquid and beads were set identical to those in the experiments. Headspace pressure was assumed to be fixed at 1 atm. The initial condition of ADM1 state variables (i.e., substrate concentrations) in the bulk liquid was estimated based on the synthetic wastewater recipe and nutrition facts of individual ingredients, which corresponds to approximately 10.8 g·L^{-1} total COD and 2.9 g·L^{-1} soluble COD. Biomass concentrations were assumed uniform across the PEG beads at the start of the simulation. And the initial relative abundance of different anaerobic microbials was derived by simulating a flow-through system with a constant influent synthetic wastewater (HRT = 4 days) for 200 days (i.e., reached steady state). The batch reactor was simulated for 8 days using default values of ADM1 kinetic and stoichiometric parameters. By adjusting the initial encapsulated biomass concentration between 3.9-7.3 g-VSS·L^{-1} (which falls below the values of maximum encapsulation density in this study), simulated cumulative CH_4 production was able to cover the range observed across different PEG beads in the experiments (**Figure S9**).

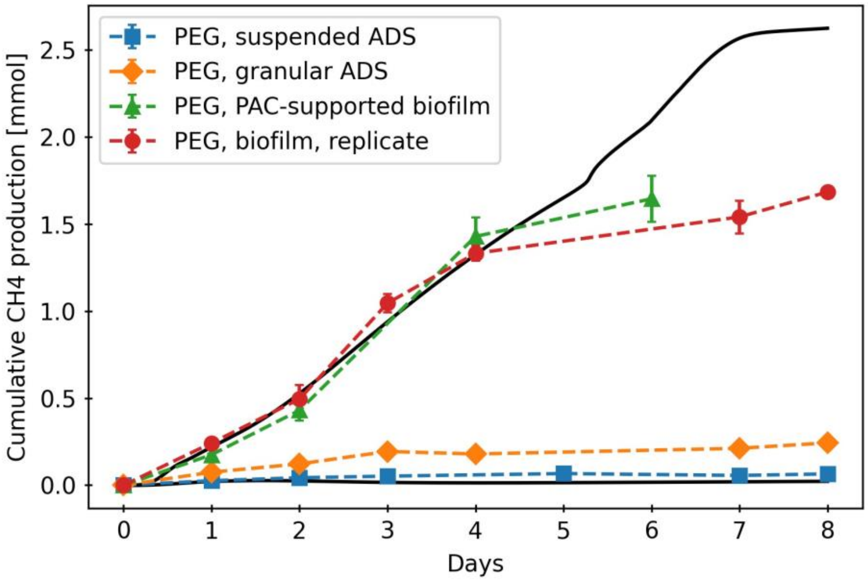

Figure S9. Simulated and measured cumulative methane production from batch experiments. Top and bottom black solid lines indicate the simulated results with high (7.3 g-VSS·L^{-1}) and low (3.9 g-VSS·L^{-1}) initial encapsulated biomass concentrations, respectively. Error bars indicate the standard deviations among triplicate batch reactors.

S2. System design

S2.1 Reactor vessel

The liquid phase volume of a reactor was determined by the specified hydraulic retention time (HRT). The headspace or the gas phase volume was assumed to be 1/10 of the liquid volume.

For upflow anaerobic sludge blanket (UASB) reactors, a minimal design upflow velocity of 0.5 m/h and a maximum depth-to-diameter ratio of 4 were used with HRT to determine the aspect ratio of the vessel. If made of concrete, fixed thickness were assumed for the cover (100 mm), the wall (150 mm), the base (160 mm), the rockwool insulation layer (25 mm), and the carbon steel facing (3 mm) of the vessel. If made of stainless steel, vessel wall thickness was determined based on its absolute internal pressure and its dimension[10,11], but the insulation thickness was assumed fixed at 50 mm. The heat transfer coefficients of the reactor vessel U [$W \cdot m^{-2} \cdot K^{-1}$] can be estimated based on the thickness and thermal conductivity of materials using the following equations.

$$\frac{1}{U} = \frac{1}{h_{water}} + \sum_j \frac{s_j}{k_j} + \frac{1}{h_{air}}$$

where h_{water} and h_{air} are convection heat transfer coefficients [$W \cdot m^{-2} \cdot K^{-1}$], s_j is the thickness of material layer j in m, k_j is the thermal conductivity of material j, in $W \cdot m^{-1} \cdot K^{-1}$. The internal structure for gas-liquid-solid separation in UASB (i.e., conical separator and baffles on vessel wall) was assumed to have a fixed aspect ratio and the size was scaled accordingly with the vessel.

Total volume of encapsulant beads in fluidized bed (FB) or packed bed (PB) reactors were determined by the HRT and specified void (or bead volume) fraction. The aspect ratio of FB and PB vessels were specified without constraint. Once the height-to-diameter ratio was determined, PB reactors would be discretized into a number of CSTRs in series for simulation. This number was estimated by rounding the height-to-diameter ratio up to the nearest integer. Excluding the three-phase separator, design algorithms for FB or PB reactor vessels were identical to those for UASB.

S2.2 Polyethylene glycol (PEG) encapsulation beads

In preliminary experiments, biomass was found to maintain good activity when encapsulated in PEG, which was among the most durable materials. The spherical encapsulation beads were manufactured through a polymerization process at ambient temperature in the laboratory. Microbials (suspended or grown on powdered activated carbon, PAC) were mixed with a solution of polyethylene glycol 1000 dimethacrylate (PEGDMA-1000, as the monomer) and N,N'-methylene-bis-acrylamide (BIS, as the crosslinking reagent) and encapsulated with the addition of N,N,N',N'-tetramethyl-ethylene-diamine (TEMED, as the crosslinking accelerator) and

ammonium persulfate (APS, as the initiator for polymerization). The detailed PEG bead manufacturing recipe used for the analyses can be found online.[12] The total mass of each ingredient was scaled with the total volume of encapsulation beads based on the yield observed in experiments.

S2.3 Hollow-fiber membrane contactor and vacuum pump

The design of hollow-fiber membrane modules for dissolved biogas extraction was based on the DuPont™ Ligasep™ degasification modules.[13] The number of membrane modules deployed was estimated by rounding the ratio between the liquid stream flowrate and the module's design flowrate up to its nearest integer. It was assumed that the membrane module requires cleaning every two weeks of continuous operation at design flowrate. The cleaning frequency and thus the amounts of NaOCl and citric acid required for cleaning were scaled nonlinearly (with a power of 0.6) with the simulated flowrate as well as TSS and TOC of the liquid stream through the module. The type, size, and heat and power utility demand of the vacuum pumps for gas extraction were estimated based on specified suction pressure, gas phase volume, and the simulated gas flowrate.[10] All design algorithms mentioned above can be accessed in the EXPOsan Github repository.[14]

S2.4 Pumping and piping

Each inlet to the reactor was equipped with a pipe and a water pump. Wastewater was assumed to be pumped into the reactor vessel from the bottom and discharged from the top. The sizing of water pumps was based on the flowrate and the total dynamic head, which includes a discharge head equivalent to the water depth in the vessel and the friction head loss through the pipe. The suction head was assumed to be negligible. The friction head loss (H_f) was estimated using the Hazen-Williams equation[15] with flowrate (q, in gal·min^{-1}), inner diameter (D, in inch), length (L), and material (i.e., the Hazen-Williams roughness coefficient c) of the pipe as inputs:

$$H_f = 2.083 \times 10^{-3} \cdot \left(\frac{100 \cdot q}{c}\right)^{1.852} \cdot ID^{-4.8655} \cdot L$$

Each influent pipe was assumed to have a length of 1.25 times the outer diameter (OD) of the reactor vessel. The length of an effluent pipe was assumed to equal reactor height $(h) + 0.25 \cdot OD$. Lengths of biogas pipes connecting the reactor headspace and biogas treatment units (i.e., iron sponge and gas holder) were assumed to be $h + OD$. The maximum inner diameter of the pipes was determined such that the resulting liquid flow velocity was at least 3 ft·s^{-1} and gas streams had a 10 ft·s^{-1} minimum velocity. This information was then used to select a standard pipe size and obtain corresponding specifications.[16,17]

For FB reactors, the recirculating wastewater flowrate is determined by the minimum fluidization velocity (u_{mf}), which is estimated by solving the following equation:[7]

$$(1-\epsilon)(\rho_b-\rho)g = 150\cdot\frac{(1-\epsilon)^2}{\epsilon^3}\cdot\frac{\mu u_{mf}}{d_b^2}+1.75\cdot\frac{1-\epsilon}{\epsilon^3}\cdot\frac{\rho u_{mf}^2}{d_b}$$

where ρ_b and ρ indicate the densities of the beads and the bulk liquid, respectively. μ is the dynamic viscosity of the bulk liquid. d_b is the diameter of the spherical beads. And ϵ indicates the void fraction of the bed prior to fluidization and assumed to be 0.4. The right-hand side of the equation represents the pressure drop per unit bed height based on the Ergun equation, which is set to equate the effective weight of the bed per unit height.

When external recirculation is needed for bead fluidization in an FB reactor, an additional pump and piping would be added to the design requirements. Sizing of the recirculation pump considers only the friction head loss through the pipe. The Ergun equation was also applied to estimate the discharge head additional to water depth for influent pump sizing for PB reactors.

S2.5 Iron sponge and gas holder

Biogas extracted or collected from all sources of the system would be treated with iron sponge to remove H_2S and other acidic gas before reuse. The cylindrical iron sponge vessel was designed based on influent H_2S concentration (assumed to be 5,000 ppmv), total gas flowrate, operating pressure and temperature, and the design contact time. [18] The vessel was assumed to be made of carbon steel (thickness = 5 mm). The iron sponge media was assumed to contain 15 lb Fe_2O_3 per bushel and have an overall density of 7,850 kg·m^{-3}, which also contains approximately 25% wood chips, 3% Na_2CO_3 and 1.5% $CaCO_3$ by weight. The iron sponge media replacement time was estimated as a function of reaction efficiency (assumed 0.7) and the influent H_2S concentration.[18] An air compressor and a control system were included for the iron sponge treatment. The design power of the air compressor was estimated as a function of the operating pressure (assumed 15 psia) and the total gas flowrate.[19]

A double-membrane gas holder was assumed for the storage of biogas before reuse, which has a slab concrete base and a dome-shape double-layer membrane cover made from polyethylene and polyvinyl chloride with varnish finish. Assuming a maximum gas holding time of 12 hours, the holding capacity and thus the construction material were estimated as a function of the total biogas flowrate.[12]

S2.6 Heat and power utilities

The electricity consumption during operation and maintenance (O&M) of the system were calculated by summing the design power (kW) across all equipment. Specifically, electricity usage for pumping wastewater (i.e., pumps), gas extraction (i.e., vacuum pumps), and air compression (i.e., compressors) were included in the estimation.

Heat energy needed to bring the influent wastewater up to the specified reaction temperature as well as to compensate for heat loss to the ambience was estimated for the design and operation of the heat exchanger. Algorithms included in BioSTEAM[20] were used for the sizing

and costing of the heat exchanger. The heat energy needed was assumed to be generated using purchased natural gas with an existing boiler onsite the brewery. Recovered biogas can partially or fully offset the natural gas purchase for heat generation. The amount of natural gas equivalent to the recovered biogas was estimated using the following equation:

$$V_{NG} = \frac{\sum_i M_i \cdot LHV_i}{C_{NG}}$$

where M_i is the mass of a biogas component i, which could be H_2, CH_4, CO_2 and water vapor. LHV_i indicates the lower heating value of the specific component, and C_{NG} is the calorific value of natural gas, which was assumed to be 39 $MJ \cdot m^{-3}$ in all simulations.

S3. Techno-economic analysis (TEA) and Life cycle assessment (LCA)

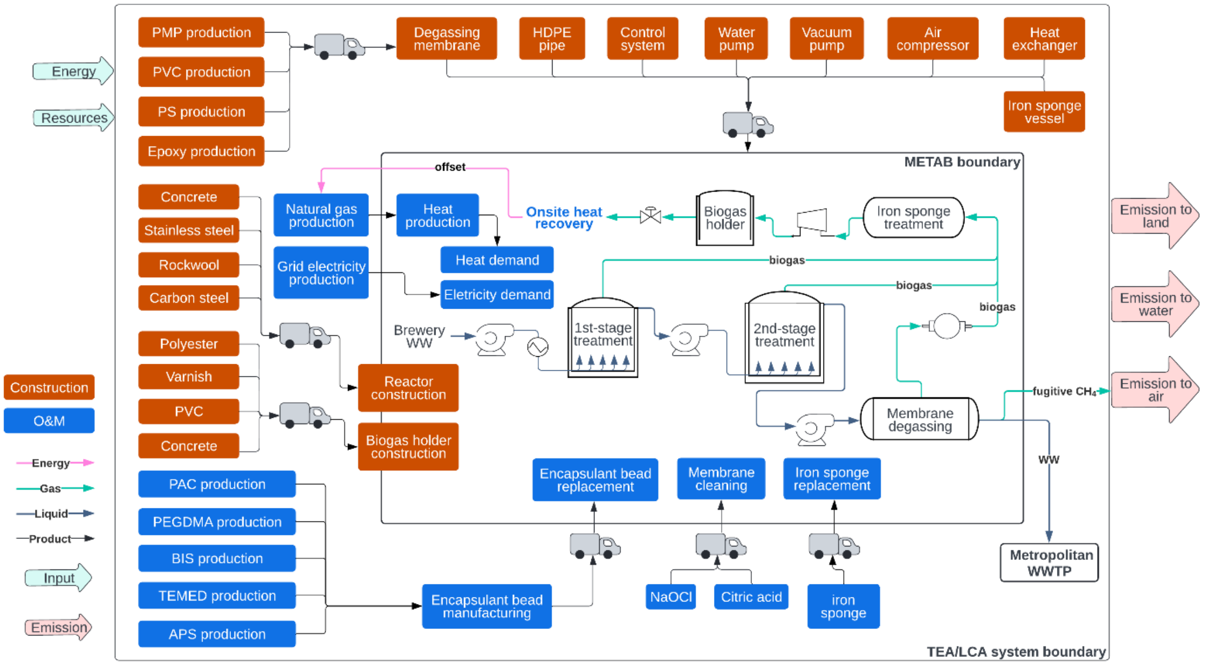

Figure S10. System boundary for TEA and LCA. METAB is an acronym for the Modular Encapsulated Two-stage Anaerobic Biological system. PMP – polymethyl pentene; PVC – polyvinylchloride; PS – polysulfone; O&M – operation and maintenance.

S3.1 Unit cost data and monetary value adjustments

Unit price for HDPE pipes (P_{HDPE}, in USD·kg^{-1}) was estimated as a function of the inner diameter (ID, in inch), which was derived using supplier data[21]:

$$P_{HDPE} = 9.625 \cdot ID^{-0.368}$$

The purchase cost of vacuum pumps for gas extraction was scaled using a power function of the total gas flowrate when estimated over 3 cfm, and this algorithm was readily available in the BioSTEAM package.[22] Otherwise, it was chosen between a 1/5-hp and a 1/3-hp commercial vacuum pumps based on the gas flowrate.[23] Similarly, the purchase cost of wastewater pumps was estimated using the existing algorithm in BioSTEAM based on its type, head, and horsepower.

These purchase costs were estimated in 2017 US dollars. Therefore, the chemical engineering plant cost index (CEPCI, 568 in 2017, 708 in 2021) was used to adjust the capital costs to 2021 USD unless specified otherwise.

Table S1. Unit costs of construction materials and O&M utilities.

Item	Price	Unit	Citation
Wall concrete†	1081.73	$\$\cdot m^{-3}$	[24]
Slab concrete†	582.48	$\$\cdot m^{-3}$	[24]
Stainless steel	1.8	$\$\cdot kg^{-1}$	[25]
Rockwool	0.59	$\$\cdot kg^{-1}$	[26]
Carbon steel	0.5	$\$\cdot kg^{-1}$	[27]
Degassing membrane	4126	$\$\cdot module^{-1}$	[28]
PEGDMA-1000	21	$\$\cdot kg^{-1}$	[29]
BIS	10	$\$\cdot kg^{-1}$	[30]
TEMED	16	$\$\cdot kg^{-1}$	[31]
APS	2.8	$\$\cdot kg^{-1}$	[32]
PAC	1.5	$\$\cdot kg^{-1}$	[33]
Iron sponge media	2.07	$\$\cdot kg^{-1}$	[24]
Iron sponge vessel, 15 psig	3110	$\$\cdot m^{-2}$	[24]
Air compressor	5180	$\$\cdot kW^{-1}$	[24]
Control system	104	$\$\cdot unit^{-1}$	[24]
Gas holder membrane	1.88	$\$\cdot m^{-2}$	[34]
Electricity	0.0913	$\$\cdot kWh^{-1}$	[35]
Natural gas*	0.85	$\$\cdot therm^{-1}$	
12.5% NaOCl solution	0.78	$·kg powder	[36]
Citric acid	0.75	$·kg	[37]

† Concrete prices were obtained in 2014 USD and adjusted to 2021 USD using the producer price index (PPI) for concrete.[38]

* Natural gas price was estimated using the 2016 gas utility bill provided by a collaborating brewery and adjusted to value in 2021 USD using the 2021-to-2016 natural gas industrial price ratio in Minnesota.[39]

S3.2 Life cycle inventory (LCI) data

Table S2. Materials and processes in the life cycle inventory.

System item	Ecoinvent 3.8 activity name
Reactor vessel, biogas pipes	market for steel, chromium steel 18/8 (GLO)
Reactor insulation	market for stone wool, packed (GLO)
Reactor vessel base, gas holder base	market for concrete slab (RoW)
Reactor vessel wall	market for concrete, medium strength (RNA)
Encapsulant production*	market for chemical factory, organics (GLO)
PEGDMA-1000 for encapsulation*,[40]	ethylene glycol production (RoW)<br>methacrylic acid production (RoW)
BIS for encapsulation*,[41]	market for polyacrylamide (GLO)<br>oxidation of methanol (GLO)<br>market for copper sulfate (GLO)<br>market for sulfuric acid (RoW)
TEMED for encapsulation*,[42]	market for dimethylamine (RoW)<br>market for ethylene dichloride (RoW)<br>market for potassium hydroxide (GLO)
PAC for encapsulation*	market for activated carbon, granular (GLO)
APS for encapsulation*	market for sodium persulfate (GLO)
DuPont™ Ligasep™ PMP fiber*,[43]	market for polypropylene, granulate (GLO)
DuPont™ Ligasep™ housing†, gas holder membrane PVC	polyvinylchloride production, suspension polymerization (RoW)<br>polyvinylchloride production, emulsion polymerization (RoW)
DuPont™ Ligasep™ potting	market for epoxy resin, liquid (RoW)
DuPont™ Ligasep™ cap and pipe	market for polysulfone (GLO)
Degassing membrane*	injection moulding (RoW)
Wastewater pipes*, degassing membrane*	extrusion, plastic pipes (RoW)
Gas holder membrane*, degassing membrane*	extrusion, plastic film (RoW)
Gas hold membrane PE	textile production, nonwoven polyester, needle-punched (IN)
Gas holder varnish	market for acrylic varnish, without water, in 87.5% solution state (RoW)
Air compressor, vacuum pump*	market for air compressor, screw-type compressor, 4kW (GLO)
Iron sponge Fe_2O_3	portafer production (RoW)
Iron sponge wood chips†	wood chips production, hardwood, at sawmill (CH)<br>wood chips production, softwood, at sawmill (RoW)
Iron sponge sodium carbonate*	market for soda ash, dense (GLO)
Iron sponge $CaCO_3$	market for calcium carbonate, precipitated (RoW)
Reactor facing, iron sponge vessel	reinforcing steel production (RoW)
Wastewater pumps	market for pump, 40W (GLO)<br>market for water pump, 22kW (GLO)<br>heat and power co-generation, oil (US-WECC)

Table S2 (cont.). Materials and processes in the life cycle inventory.

System item	Ecoinvent 3.8 activity name
Wastewater pipe manufacturing*†, encapsulant production*†	heat and power co-generation, natural gas, conventional power plant, 100MW electrical (IE)
Wastewater pipes	market for polyethylene, high density, granulate (GLO)
	market for plastic processing factory (GLO)
Wastewater pipe manufacturing*, degassing membrane manufacturing*, encapsulant production*, O&M electricity	electricity, high voltage, production mix (US-WECC)
Onsite heat generation†	heat production, natural gas, at boiler modulating <100kW (RoW)
	heat production, natural gas, at boiler condensing modulating <100kW (RoW)
	heat production, natural gas, at boiler atmospheric non-modulating <100kW (RoW)
	heat production, natural gas, at boiler fan burner non-modulating <100kW (RoW)
	heat production, natural gas, at boiler atm. low-NOx condensing non-modulating <100kW (RoW)
	heat production, natural gas, at boiler fan burner low-NOx non-modulating <100kW (RoW)
	heat production, natural gas, at boiler atmospheric low-NOx non-modulating <100kW (RoW)
Transportation of encapsulant beads†	transport, freight, lorry 3.5-7.5 metric ton, EURO3 (RoW)
	transport, freight, lorry 3.5-7.5 metric ton, EURO4 (RoW)
	transport, freight, lorry 3.5-7.5 metric ton, EURO5 (RoW)
	transport, freight, lorry 3.5-7.5 metric ton, EURO6 (RoW)
Natural gas offset by recovered biogas	market for natural gas, low pressure (RoW)
NaOCl for membrane cleaning	sodium hypochlorite production, product in 15% solution state (RoW)
Citric acid for membrane cleaning	market for citric acid (GLO)

* Inventory data were not directly available for the item and a surrogate item or activities identified in the upstream production processes (citations included) were used as replacement. Direct emission of fugitive CH_4 was also included in the life cycle inventory.

† Multiple activities were averaged due to lack of information to choose a specific activity.

S4. Discrete design decision analysis settings

Discrete combinations of decision variables (DVs) 0-3 in **Figure S11** yield 12 distinct system configurations. The ranges of the continuous DVs were chosen based on a combination of the existing experimental setup, the pilot system at the brewery,[44] and preliminary simulation results such that the resulting decision space was feasible and broad enough to enclose the optimal decision region.

Only two-stage systems considered applying active H_2 extraction from the first stage. And only the first stage was subjected to temperature control. The pH of the first and second stages were controlled at 5.8 and 7.2, respectively. The ratio of HRT between the first and the second stages was fixed at 1:11. Single-stage systems' pH was controlled at 6.5. When sidestream membrane H_2 extraction was applied to a packed bed reactor, the hydrodynamic of the reactor was assumed to resemble a CSTR regardless of its aspect ratio.

Different values of kinetic parameters were used for 22°C and 35°C reactions (**Table S4**). Specifically, default mesophilic biological reaction rate constants in ADM1 were used when temperature was set at 35°C. Rate constants at 22°C were assumed to be fractions of those at 35°C. The overall liquid-gas transfer coefficient $k_L a$ [d^{-1}] is also dependent on temperature T [K] in simulation following equation:

$$k_L a = 0.56 \cdot T + 27.9$$

Other kinetic parameters for liquid-gas transfer processes and acid-base reactions were temperature-corrected using the Van 't Hoff equation as described in ADM1.

Table S3. A constant brewery wastewater composition used in all simulations, expressed in ADM1 state variables. Values are 1×10^{-3} kg COD·m^{-3} for all biomass state variables.

Variable	Component	Value	Unit
S_{su}	Sugar	3.0	kg COD·m^{-3}
S_{aa}	Amino acids	0.6	kg COD·m^{-3}
S_{fa}	Long-chain fatty acids	0.4	kg COD·m^{-3}
S_{va}	Valerate	0.4	kg COD·m^{-3}
S_{bu}	Butyrate	0.4	kg COD·m^{-3}
S_{pro}	Propionate	0.4	kg COD·m^{-3}
S_{ac}	Acetate	0.4	kg COD·m^{-3}
S_{h2}	Dissolved hydrogen	5×10^{-9}	kg COD·m^{-3}
S_{ch4}	Dissolved methane	5×10^{-6}	kg COD·m^{-3}
S_{IC}	Soluble inorganic carbon	0.04	kmol C·m^{-3}
S_{IN}	Soluble inorganic nitrogen	0.01	kmol N·m^{-3}
S_I	Inert solubles	0.02	kg COD·m^{-3}
X_c	Complex particulates	0.1	kg COD·m^{-3}
X_{ch}	Carbohydrates	0.3	kg COD·m^{-3}
X_{pr}	Proteins	0.5	kg COD·m^{-3}
X_{li}	Lipids	0.25	kg COD·m^{-3}
X_I	Inert particulates	0.025	kg COD·m^{-3}
S_{cat}	Cations	0.04	kmol·m^{-3}
S_{an}	Anions	0.02	kmol·m^{-3}

Table S4. Values of biological reaction rate constants at different temperatures in simulation.

Rate constants	Processes	Unit	Value at 35°C (ADM1 default)[45]	Value at 22°C*	Citation
k_{dis}	Disintegration	d^{-1}	0.5	0.15	a
$k_{hyd,ch}$	Carbohydrate hydrolysis	d^{-1}	10	3	46
$k_{hyd,pr}$	Protein hydrolysis	d^{-1}	10	3	46
$k_{hyd,li}$	Lipid hydrolysis	d^{-1}	10	3	46
k_{su}	Sugar uptake	$COD \cdot COD^{-1} \cdot d^{-1}$	30	6.43	b
k_{aa}	Amino acid uptake	$COD \cdot COD^{-1} \cdot d^{-1}$	50	10.7	b
k_{fa}	LCFA uptake	$COD \cdot COD^{-1} \cdot d^{-1}$	6	1.29	47
k_{c4}	Butyrate or valerate uptake	$COD \cdot COD^{-1} \cdot d^{-1}$	20	12	47
k_{pro}	Propionate uptake	$COD \cdot COD^{-1} \cdot d^{-1}$	13	8.32	47
k_{ac}	Acetate uptake	$COD \cdot COD^{-1} \cdot d^{-1}$	8	3.94	47
k_{h2}	H_2 uptake	$COD \cdot COD^{-1} \cdot d^{-1}$	35	17.2	c
k_{dec}	Biomass decay	d^{-1}	0.02	0.02	48

* Values at 22°C were derived by multiplying the value at 35°C by a fraction obtained from the citation.
[a] Assumed same fraction as the hydrolysis processes.
[b] Assumed same fraction as LCFA (long-chain fatty acids) uptake.
[c] Assumed same fraction as acetate uptake.

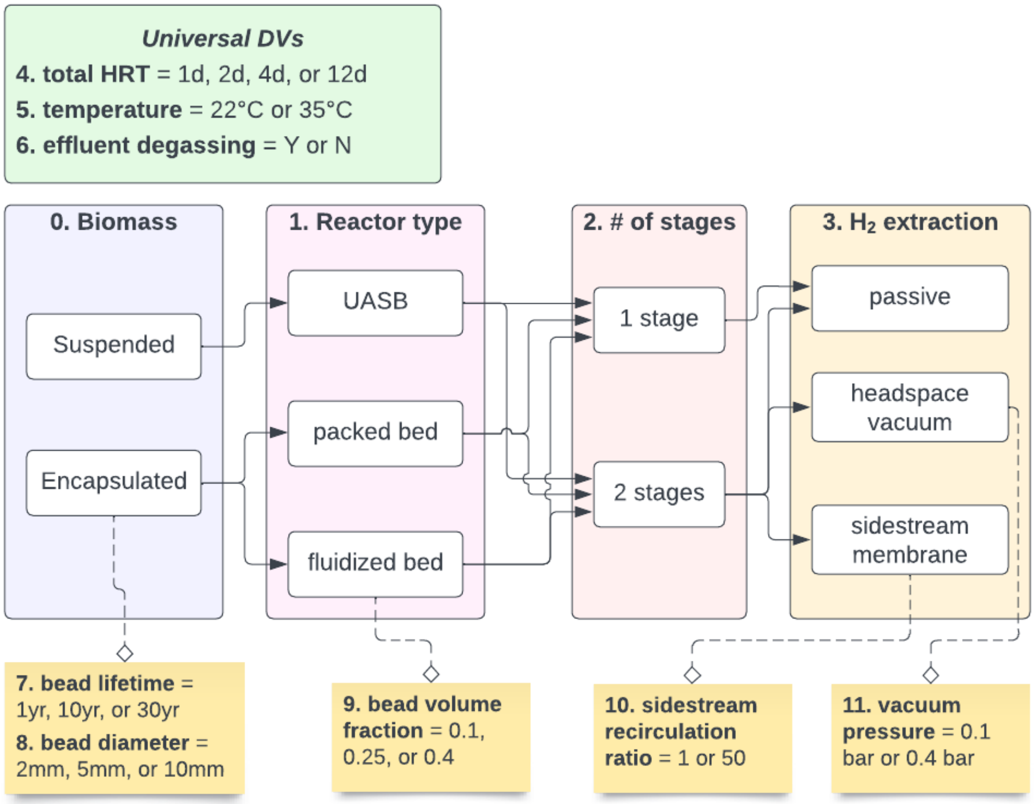

Figure S11. The 11 design decision variables (DVs) and their discrete values in the discrete decision analysis.[49]

S5. Monte Carlo simulation settings

Table S5. Parameters and their uncertainty in Monte Carlo simulation.

Parameter	Unit	Distribution	Values[b]	Citation
ADM1[a]				
k_{dis}	d^{-1}	Triangular	0.001, 0.150, 0.600	[45,46,48]
$k_{hyd,ch}$	d^{-1}	Triangular	0.1, 3.0, 3.0	[45,46,48]
k_{ac}	$COD \cdot COD^{-1} \cdot d^{-1}$	Triangular	3.94, 3.94, 15.75	[45,47,48]
k_{h2}	$COD \cdot COD^{-1} \cdot d^{-1}$	Triangular	2.00, 3.94, 4.92	[45,47,48]
K_{ac}	$kgCOD \cdot m^{-3}$	Triangular	0.05, 0.15, 0.60	[45,48]
K_{h2}	$kgCOD \cdot m^{-3}$	Triangular	1.0e-6, 7.0e-6, 2.8e-5	[45,48]
Membrane				
Degassing efficiency factor	-	Uniform	0, 1	[c]
System				
HRT	h	Uniform	4, 120	[d]
UASB				
Solid retention efficacy	-	Triangular	0.870, 0.963, 0.995	[50]
Encapsulation				
Maximum encapsulation density	$gTSS \cdot L^{-1}$	Log-uniform	11, 22	[51]
Bead density	$kg \cdot m^{-3}$	Uniform	990, 1860	[51]
Bead-to-water diffusivity ratio	-	Triangular	0.20, 0.55, 1.10	[52,53]
Bead lifetime	yr	Uniform	1, 10	[54]
Bead diameter	mm	Uniform	1, 5	[55–58, d]
Fluidized bed				
Bead volume fraction	-	Uniform	0.03, 0.25	[e]
Reactor height-to-diameter ratio	-	Uniform	0.5, 2.0	[e]
Packed bed				
Bed void fraction	-	Triangular	0.35, 0.39, 0.45	[59–61]
Reactor height-to-diameter ratio	-	Uniform	1, 5	[e]

[a] Only the most frequently modified parameters (i.e., those for hydrolysis and methanogenesis) are included in uncertainty analysis.[48]

[b] Probability density functions were characterized by minimum and maximum values (uniform and log-uniform) or minimum, mode, and maximum values (triangular).

[c] Degassing efficiency factor was used to scale the removal efficiencies of biogas species monotonically between their minimum (55%, 36%, 6% for H_2, CH_4, and CO_2 respectively) and maximum (70%, 55%, 20%) values measured in this study[49].

[d] Upper bound was determined based on results from the discrete design decision analysis.

[e] Values were assumed based on results from the discrete design decision analysis.

S6. Additional results

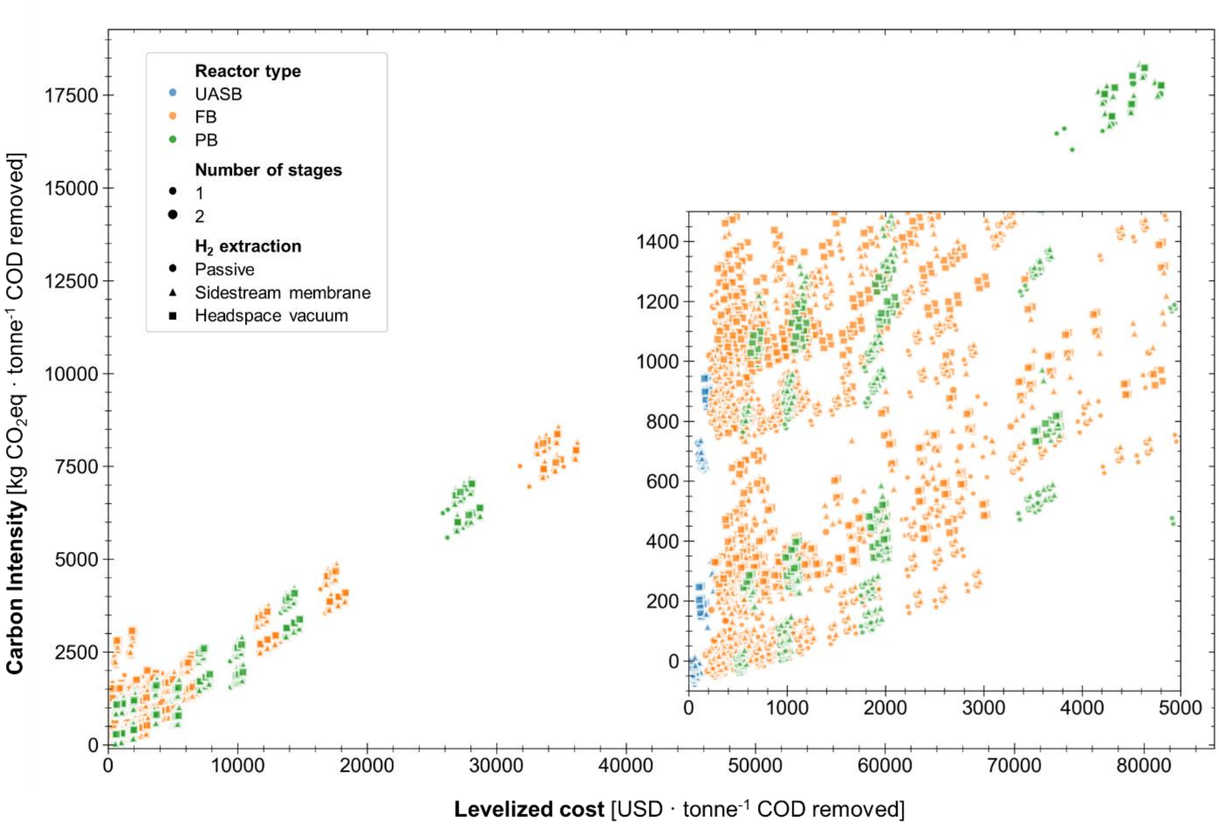

Figure S12. Simulated levelized cost and carbon intensity of COD removal for 3,552 discrete combinations of design decisions. Each data point in the plot represents a distinct combination.

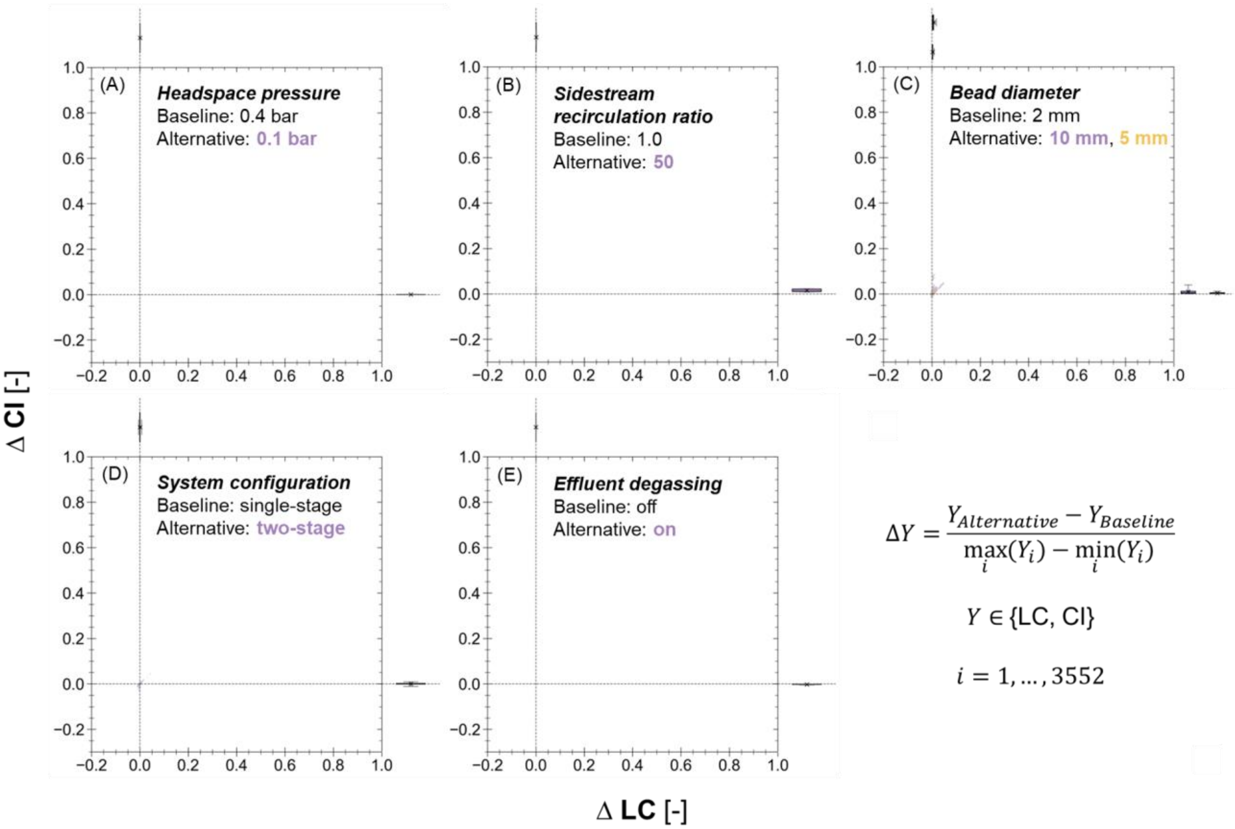

$$\Delta Y = \frac{Y_{Alternative} - Y_{Baseline}}{\max_i(Y_i) - \min_i(Y_i)}$$

$$Y \in \{\text{LC, CI}\}$$

$$i = 1, \ldots, 3552$$

Figure S13. Relative impacts of individual design decisions on levelized cost and carbon intensity of COD removal quantified by pair wise comparison. Most data points are tightly clustered around the origin, and as a result, the 2D kernel distributions are hardly visible.

Table S6. Spearman's rank correlation coefficient between fluidized bed systems' indicator values obtained from Monte Carlo simulation. All coefficients have $p < 2\times10^{-16}$.

		w/ effluent degassing		w/o effluent degassing	
		LC	CI	LC	CI
w/ effluent degassing	LC	1	0.839	0.997	0.753
	CI		1	0.804	0.980
w/o effluent degassing	LC			1	0.713
	CI				1

Table S7. Spearman's rank correlation coefficient between packed bed systems' indicator values obtained from Monte Carlo simulation. All coefficients have $p < 2\times10^{-16}$.

		w/ effluent degassing		w/o effluent degassing	
		LC	CI	LC	CI
w/ effluent degassing	LC	1	0.992	1.000	0.991
	CI		1	0.992	0.999
w/o effluent degassing	LC			1	0.991
	CI				1

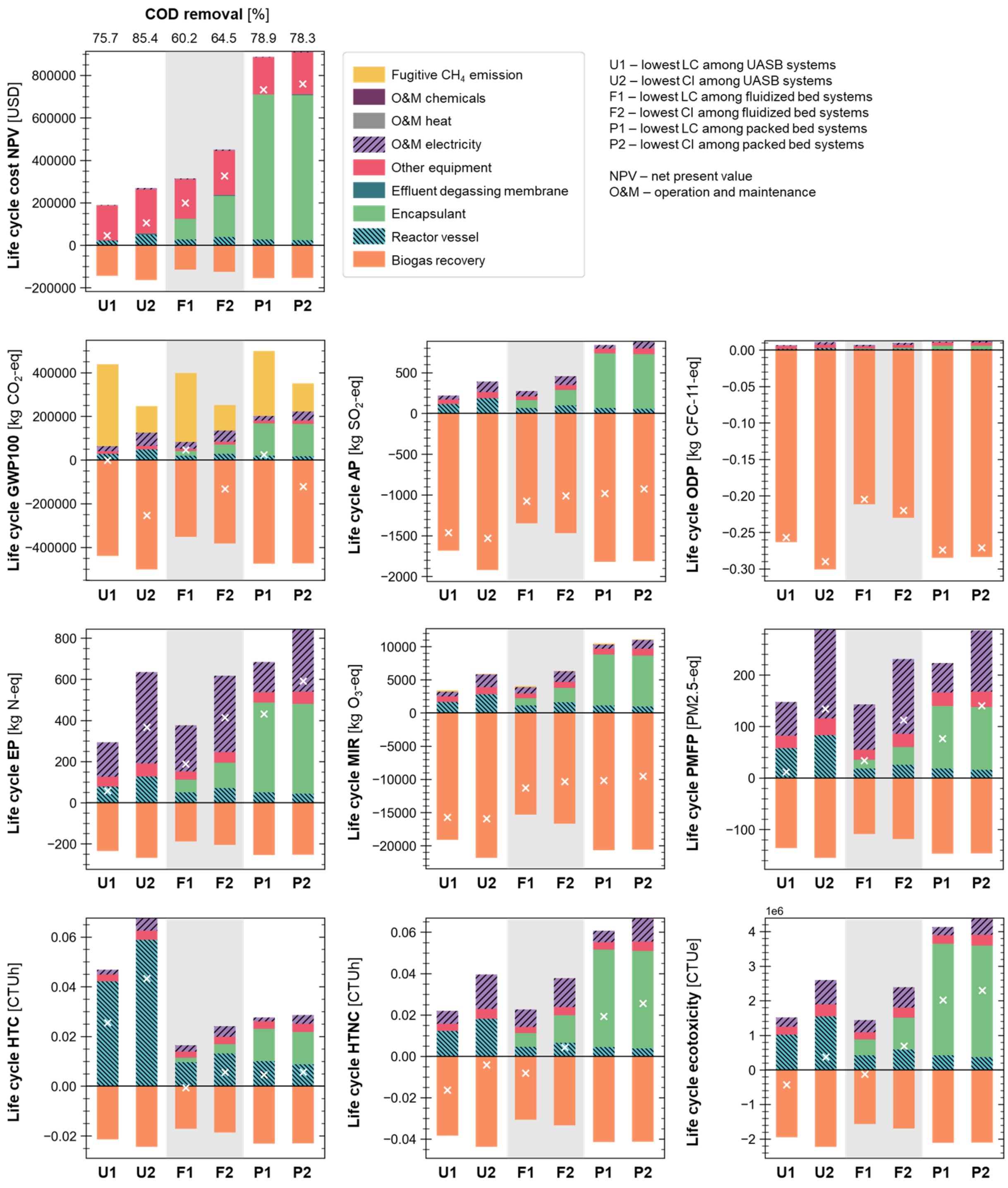

Figure S14. Attributing the life cycle cost and environmental impacts to different sources for the best performing suspended growth and encapsulated configurations in the discrete decision analysis. The white cross markers indicate the net life cycle cost or impact. Ambient-temperature operation and passive biogas collection are seen across all best performing configurations. Across reactor types, systems with the lowest LC (i.e., U1, F1, P1) consistently employ a two-stage configuration without effluent degasification. But those with the lowest CI (i.e., U2, F2, P2) are all single-stage systems with effluent degasification.

Figure S14 (cont.). Best encapsulated systems (i.e., F1, F2, P1, P2) commonly use 2mm beads of 30-yr longevity. F1 and F2 both have a bead volume fraction of 10%. Both P1 and P2 have a 1-d HRT. But for UASB or fluidized bed systems, moderate HRT (4d) tends to yield the lowest CI whereas short HRTs (1d for U1, 2d for F1) yield lowest LCs.

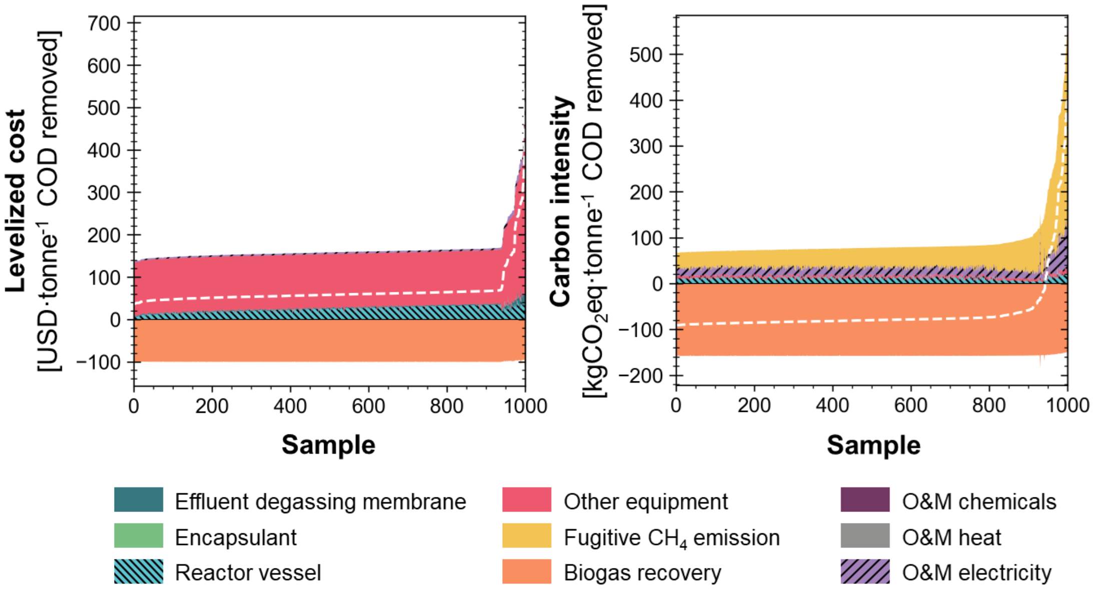

Figure S15. Breakdowns of the simulated levelized cost and carbon intensity of COD removal by UASB systems (with effluent degassing). The samples are sorted in ascending order of metric values for better visualization and thus the x-axis value does not imply the actual order of simulation. White dashed lines indicate the net LC or CI of COD removal.

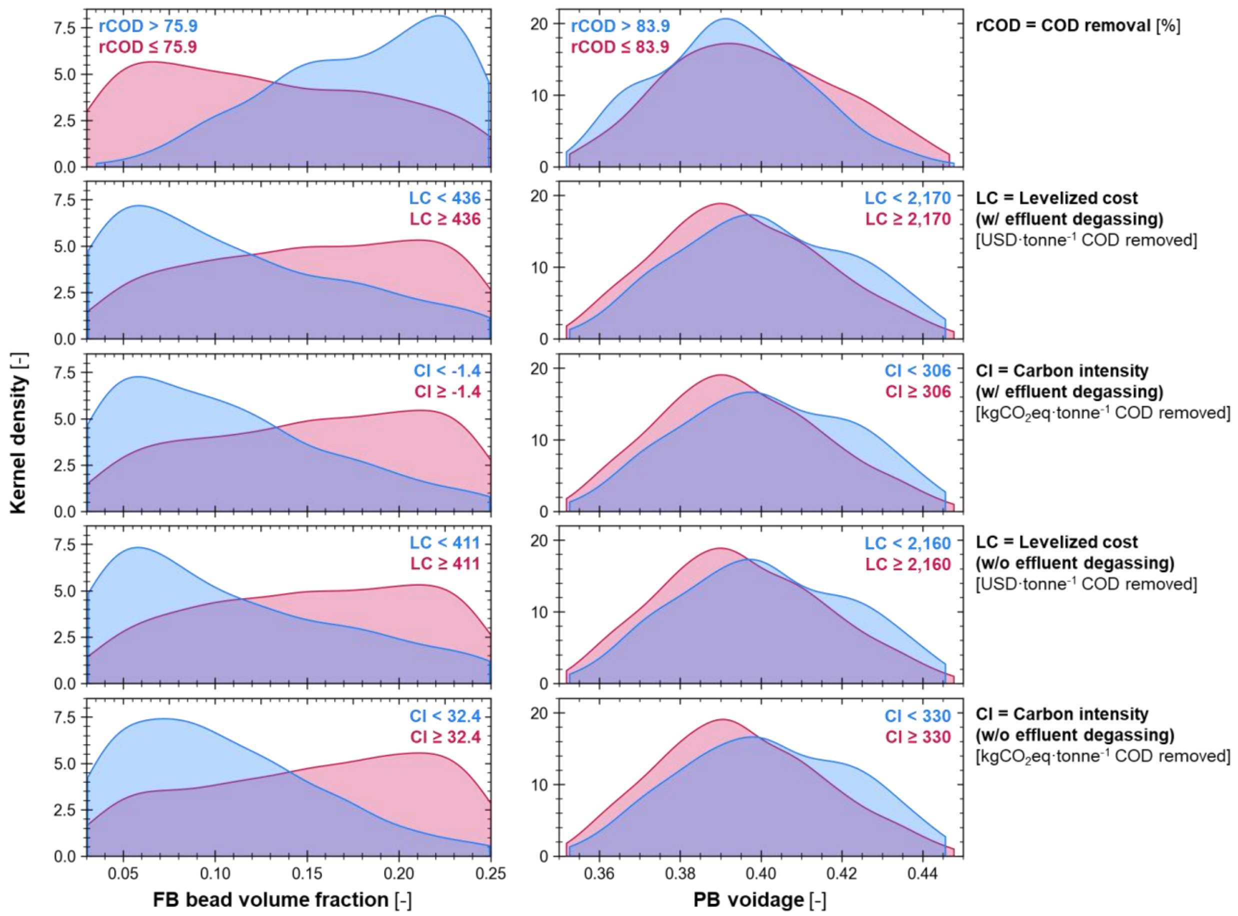

Figure S16. Kernel density plots of parameter distributions by "good" vs. "bad" indicator values of the encapsulated systems. The good and bad groups account for 25% and 75% of all samples, respectively.

References

(1) Batstone, D. J.; Keller, J.; Angelidaki, I.; Kalyuzhnyi, S. V.; Pavlostathis, S. G.; Rozzi, A.; Sanders, W. T. M.; Siegrist, H.; Vavilin, V. A. The IWA Anaerobic Digestion Model No 1 (ADM1). *Water Science and Technology* **2002**, *45* (10), 65–73. https://doi.org/10.2166/wst.2002.0292.

(2) Rosén, C.; Jeppsson, U. *Aspects on ADM1 Implementation within the BSM2 Framework*; Lund, 2005.

(3) Gernaey, K. V.; Jeppsson, U.; Vanrolleghem, P. A.; Copp, J. B. *Benchmarking of Control Strategies for Wastewater Treatment Plants*; IWA Publishing: London, 2014. https://doi.org/10.2166/9781780401171.

(4) Schiesser, W. E.; Griffiths, G. W. An Introduction to the Method of Lines. In *A Compendium of Partial Differential Equation Models*; Cambridge University Press, 2009; pp 1–17. https://doi.org/10.1017/CBO9780511576270.002.

(5) Gutenberger, G. M. Polyethylene Glycol as a Robust, Biocompatible Encapsulant for Two-Stage Treatment of Food and Beverage Wastewater. M.S. thesis, University of Minnesota, 2023. https://hdl.handle.net/11299/256968 (accessed 2023-11-26).

(6) Hydromantis. *GPS-X Technical Reference - v8.0*. https://www.hydromantis.com/help/GPS-X/docs/8.0/Technical/index.html (accessed 2023-05-04).

(7) Richardson, J. F.; Harker, J. H.; Backhurst, J. R. Fluidisation. In *Chemical Engineering*; Butterworth-Heinemann, 2002; Vol. 2, pp 291–371. https://doi.org/10.1016/B978-0-08-049064-9.50017-5.

(8) Gutenberger, G. M.; Holgate, O. M.; Arnold, W. A.; Guest, J. S.; Novak, P. J. *Polyethylene Glycol as a Robust, Biocompatible Encapsulant for Two-Stage Treatment of Food and Beverage Wastewater*; 2023.

(9) Zhu, K.; Davis, C. W.; Novak, P. J.; Arnold, W. A. Effects of Encapsulation on the Chemical Inhibition of Anaerobic Hydrogen- and Methane-Producing Microbial Cells. *Bioresour Technol Rep* **2020**, *11*, 100451. https://doi.org/10.1016/J.BITEB.2020.100451.

(10) Seider, W. D.; Lewin, D. R.; Seader, J. D.; Widagdo, S.; Gani, R.; Ng, K. M. *Product and Process Design Principles: Synthesis, Analysis and Evaluation*, 4th ed.; John Wiley & Sons Inc.: New York, 2017.

(11) BioSTEAM Development Group. *flash_vessel_design*. https://github.com/BioSTEAMDevelopmentGroup/biosteam/blob/master/biosteam/units/design_tools/flash_vessel_design.py (accessed 2023-05-03).

(12) Quantitative Sustainable Design Group. *EXPOsan/equipment*. https://github.com/QSD-Group/EXPOsan/blob/main/exposan/metab/equipment.py (accessed 2023-05-03).

(13) DuPont. DuPont™ Ligasep™ Degasification Modules. 2020. https://www.lenntech.com/Data-sheets/DuPont-UF-Ligasep-LDM-040-L.pdf (accessed 2023-05-03).

(14) Quantitative Sustainable Design Group. *EXPOsan/exposan/metab*. https://github.com/QSD-Group/EXPOsan/tree/main/exposan/metab (accessed 2023-05-03).

(15) Williams, G. S.; Hazen, A. *Hydraulic Tables: Showing the Loss of Head Due to the Friction of Water Flowing in Pipes, Aqueducts, Sewers, Etc. and the Discharge over Weirs.*; J. Wiley, 1905.

(16) American Piping Products. *Pipe Dimension Chart*. https://amerpipe.com/wp-content/uploads/2015/10/APP-chart-v7-web.pdf (accessed 2023-05-03).

(17) Piping Designer. *HDPE, ANSI DR 11.0, 200 PSI, IPS (in)*. https://www.piping-designer.com/index.php/datasheets/piping-datasheets/1651-pipe-hdpe-ansi-dr-11-0-ips-in (accessed 2023-05-03).

(18) Sheldford, T.; Gooch, C. Iron Sponge Design Considerations: Vessel Sizing. In *HYDROGEN SULFIDE REMOVAL FROM BIOGAS* ; 2017; Vol. Part 3B.

(19) Engineering ToolBox. *Horsepower required to Compress Air*. https://www.engineeringtoolbox.com/horsepower-compressed-air-d_1363.html (accessed 2023-05-03).

(20) Cortes-Peña, Y.; Kumar, D.; Singh, V.; Guest, J. S. BioSTEAM: A Fast and Flexible Platform for the Design, Simulation, and Techno-Economic Analysis of Biorefineries under Uncertainty. *ACS Sustain Chem Eng* **2020**, *8* (8), 3302–3310. https://doi.org/10.1021/acssuschemeng.9b07040.

(21) Hdpe Supply. *HDPE Straight Length Pipe*. https://hdpesupply.com/hdpe-straight-length-pipe/ (accessed 2023-05-03).

(22) BioSTEAM Development Group. *biosteam/vacuum*. https://github.com/BioSTEAMDevelopmentGroup/biosteam/blob/master/biosteam/units/design_tools/vacuum.py (accessed 2023-05-03).

(23) Robinair. *Vacuum Pumps & Gauges*. https://www.amazon.com/stores/page/D93FDADF-4140-467A-92A3-751750064722?ingress=2&visitId=1d2a88aa-ecda-43a9-a273-d2917f91b76c&ref_=ast_bln (accessed 2023-05-03).

(24) Hydromantis. CapdetWorks 4.0. https://www.hydromantis.com/CapdetWorks.html (accessed 2023-05-03).

(25) Alibaba. *Brushed Stainless Steel Plate 304*. https://www.alibaba.com/product-detail/brushed-stainless-steel-plate-304l-stainless_1600391656401.html?spm=a2700.details.0.0.230e67e6IKwwFd (accessed 2023-05-03).

(26) Alibaba. *Mineral Wool Insulation*. https://www.alibaba.com/product-detail/mineral-wool-insulation-price-mineral-wool_60101640303.html?spm=a2700.7724857.0.0.262334d1rZXb48 (accessed 2023-05-03).

(27) Alibaba. *Carbon Steel Hot Rolled*. https://www.alibaba.com/product-detail/ASTM-A106-Ss400-Q235-Standard-Ms_1600406694387.html?s=p (accessed 2023-05-03).

(28) DWS Advantage. *LigaSep™ LDM 040 LS ANSI*. https://www.dwsadvantage.com/us/ligasep-ldm-040-ls-ansi-na.html (accessed 2023-05-03).

(29) ECHEMI. *Poly(ethyleneglycol)dimethacrylate 99% Colourless liquid C8H4Na2O4 Pharmacy Grade Senwayer Pharmacy Grade from Wuhan Senwayer Century Chemical Co.,Ltd - ECHEMI*. https://www.echemi.com/produce/pr2210112485-polyethyleneglycoldimethacrylate-99-colourless-liquid-c8h4na2o4-pharmacy-grade-senwayer.html (accessed 2023-05-03).

(30) Alibaba. *N,N'-methylenebisacrylamide Cas 110-26-9 With High Quality*. https://www.alibaba.com/product-detail/Best-price-N-N-Methylenebisacrylamide-CAS_1600724924581.html?spm=a2700.galleryofferlist.normal_offer.d_title.58d341d0GVNAh1 (accessed 2023-05-03).

(31) Alibaba. *Crosslinking Catalyst Tetramethylethylenediamine TEMED*. https://www.alibaba.com/product-detail/Crosslinking-catalyst-Tetramethylethylenediamine-TEMED_1600273096288.html?spm=a2700.galleryofferlist.normal_offer.d_title.73077f226sNObF (accessed 2023-05-03).

(32) Alibaba. *Ammonium Persulfate Industrial Grade*. https://www.alibaba.com/product-detail/Persulfate-Ammonium-Molecular-formula-NH4-2S2O8_1600618995452.html?spm=a2700.galleryofferlist.normal_offer.d_title.17b42c417WytQh (accessed 2023-05-03).

(33) Alibaba. *325mesh Wood Based Powder Activated Carbon*. https://www.alibaba.com/product-detail/Best-Sale-325mesh-Wood-Based-Powder_1600694829290.html?spm=a2700.galleryofferlist.normal_offer.d_title.62c1efdbTj3aHI&s=p (accessed 2023-05-03).

(34) Alibaba. *1.2mm Pvc Waterproof Membrane*. https://www.alibaba.com/product-detail/Smooth-PVC-side-wall-underground-waterproofing_1600772195391.html?spm=a2700.galleryofferlist.normal_offer.d_title.7a75527fAzN9FP (accessed 2023-05-03).

(35) U.S. Energy Information Administration. *2021 Utility Bundled Sales to Ultimate Customers-Industrial (Data from Forms EIA-861-Schedules 4A & 4D and EIA-861S)*; 2022. https://www.eia.gov/electricity/sales_revenue_price/pdf/table8.pdf (accessed 2023-05-03).

(36) Alibaba. *Sodium Hypochlorite Powder*. https://www.alibaba.com/product-detail/Sodium-Hypochlorite-Price-sodium-Hypochlorite-Production_1600829470036.html?spm=a2700.galleryofferlist.normal_offer.d_title.253d4c6dXy4FXN (accessed 2023-05-03).

(37) Alibaba. *Powder Lemon Acid*. https://www.alibaba.com/product-detail/Best-selling-powder-lemon-acid-price_1600657054188.html?spm=a2700.galleryofferlist.0.0.7141505dSfKzKN (accessed 2023-05-03).

(38) Statista. *PPI of concrete products in U.S. 1926-2022*. https://www.statista.com/statistics/195544/us-producer-price-index-of-concrete-products-since-1990/ (accessed 2023-05-03).

(39) U.S. Energy Information Administration. *Minnesota Natural Gas Industrial Price*. https://www.eia.gov/dnav/ng/hist/n3035mn3a.htm (accessed 2023-05-03).

(40) PubChem. *Glycol Dimethacrylate*. https://doi.org/10.1038/SDATA.2018.125.

(41) Lundberg, L. A. Alkylidene-Bis-Acrylamides. US2475846A, July 12, 1949. https://patents.google.com/patent/US2475846A/en?oq=2475846 (accessed 2023-05-03).

(42) Hammerstrom, K.; Spielberger, G. Process for the Continuous Preparation of Tetramethylethylene-Diamine. US4053516A, October 11, 1977.

(43) Lopez, L. C.; Wilkes, G. L.; Stricklen, P. M.; White, S. A. Synthesis, Structure, and Properties of Poly(4-Methyl-1 -Pentene). *Journal of Macromolecular Science, Part C* **2006**, *32* (3–4), 301–406. https://doi.org/10.1080/15321799208021429.

(44) Chen, S.; Arnold, W.; Wright, N.; Zhu, K.; Ajayi, O.; Novak, P. Encapsulation Technology for Decentralized Brewery Wastewater Treatment: A Small Pilot Experiment. *Bioresour Technol* **2022**, *347*, 126435. https://doi.org/10.1016/j.biortech.2021.126435.

(45) IWA Task Group for Mathematical Modelling of Anaerobic Digestion Processes. *Anaerobic Digestion Model No.1 (ADM1)*; IWA Publishing, 2005. https://doi.org/10.2166/9781780403052.

(46) Donoso-Bravo, A.; Retamal, C.; Carballa, M.; Ruiz-Filippi, G.; Chamy, R. Influence of Temperature on the Hydrolysis, Acidogenesis and Methanogenesis in Mesophilic Anaerobic Digestion: Parameter Identification and Modeling Application. *Water Science and Technology* **2009**, *60* (1), 9–17. https://doi.org/10.2166/wst.2009.316.

(47) Rebac, S.; Ruskova, J.; Gerbens, S.; van Lier, J. B.; Stams, A. J. M.; Lettinga, G. High-Rate Anaerobic Treatment of Wastewater under Psychrophilic Conditions. *J Ferment Bioeng* **1995**, *80* (5), 499–506. https://doi.org/10.1016/0922-338X(96)80926-3.

(48) Mo, R.; Guo, W.; Batstone, D.; Makinia, J.; Li, Y. Modifications to the Anaerobic Digestion Model No. 1 (ADM1) for Enhanced Understanding and Application of the Anaerobic Treatment Processes – A Comprehensive Review. *Water Res* **2023**, *244*, 120504. https://doi.org/10.1016/j.watres.2023.120504.

(49) Song, I.; Soulia, P. S.; Novak, P. J.; Arnold, W. A.; Wright, N. C. Dissolved Gas Recovery from Water Using a Sidestream Hollow-Fiber Membrane Module: First Principles Model Synthesis and Steady-State Validation. *J Memb Sci* **2024**, *689*, 122134. https://doi.org/10.1016/J.MEMSCI.2023.122134.

(50) Hao, F. L.; Shen, M. W. Development, Simulation, and Laboratory Test of Novel Gas-Solid-Liquid Separator for UASB/EGSB Reactor of Wastewater Treatment. *J Environ Chem Eng* **2021**, *9* (3), 105217. https://doi.org/10.1016/J.JECE.2021.105217.

(51) Singh, L.; Siddiqui, M. F.; Ahmad, A.; Rahim, M. H. A.; Sakinah, M.; Wahid, Z. A. Biohydrogen Production from Palm Oil Mill Effluent Using Immobilized Mixed Culture. *Journal of Industrial and Engineering Chemistry* **2013**, *19* (2), 659–664. https://doi.org/10.1016/J.JIEC.2012.10.001.

(52) Offeddu, G. S.; Axpe, E.; Harley, B. A. C.; Oyen, M. L. Relationship between Permeability and Diffusivity in Polyethylene Glycol Hydrogels. *AIP Adv* **2018**, *8* (10), 105006. https://doi.org/10.1063/1.5036999.

(53) Richbourg, N. R.; Peppas, N. A. Solute Diffusion and Partitioning in Multi-Arm Poly(Ethylene Glycol) Hydrogels. *J Mater Chem B* **2023**, *11* (2), 377–388. https://doi.org/10.1039/D2TB02004A.

(54) Isaka, K.; Kimura, Y.; Osaka, T.; Tsuneda, S. High-Rate Denitrification Using Polyethylene Glycol Gel Carriers Entrapping Heterotrophic Denitrifying Bacteria. *Water Res* **2012**, *46* (16), 4941–4948. https://doi.org/10.1016/j.watres.2012.05.050.

(55) Cristina, G.; Camelin, E.; Ottone, C.; Fraterrigo Garofalo, S.; Jorquera, L.; Castro, M.; Fino, D.; Schiappacasse, M. C.; Tommasi, T. Recovery of Humic Acids from Anaerobic Sewage Sludge: Extraction, Characterization and Encapsulation in Alginate Beads. *Int J Biol Macromol* **2020**, *164*, 277–285. https://doi.org/10.1016/j.ijbiomac.2020.07.097.

(56) Covarrubias, S. A.; de-Bashan, L. E.; Moreno, M.; Bashan, Y. Alginate Beads Provide a Beneficial Physical Barrier against Native Microorganisms in Wastewater Treated with Immobilized Bacteria and Microalgae. *Appl Microbiol Biotechnol* **2012**, *93* (6), 2669–2680. https://doi.org/10.1007/s00253-011-3585-8.

(57) Bustos-Terrones, Y. A.; Bandala, E. R.; Moeller-Chávez, G. E.; Bustos-Terrones, V. Enhanced Biological Wastewater Treatment Using Sodium Alginate-Immobilized Microorganisms in a Fluidized Bed Reactor. *Water Science and Engineering* **2022**, *15* (2), 125–133. https://doi.org/10.1016/j.wse.2022.02.002.

(58) Zhang, Y.; Hui, B.; Ye, L. Reactive Toughening of Polyvinyl Alcohol Hydrogel and Its Wastewater Treatment Performance by Immobilization of Microorganisms. *RSC Adv* **2015**, *5* (111), 91414–91422. https://doi.org/10.1039/C5RA20495J.

(59) Foumeny, E. A.; Benyahia, F. Predictive Characterization of Mean Voidage in Packed Beds. *Heat Recovery Systems and CHP* **1991**, *11* (2–3), 127–130. https://doi.org/10.1016/0890-4332(91)90126-O.

(60) Benyahia, F.; O'Neill, K. E. Enhanced Voidage Correlations for Packed Beds of Various Particle Shapes and Sizes. *Particulate Science and Technology* **2007**, *23* (2), 169–177. https://doi.org/10.1080/02726350590922242.

(61) Reyes, S. C.; Iglesia, E. Monte Carlo Simulations of Structural Properties of Packed Beds. *Chem Eng Sci* **1991**, *46* (4), 1089–1099. https://doi.org/10.1016/0009-2509(91)85102-4.
